# Hydrophobic residues in the α‐synuclein NAC domain drive seed‐competent fibril formation and are targeted by peptide inhibitors

**DOI:** 10.1111/febs.70222

**Published:** 2025-08-17

**Authors:** Viswanath Das, Sayed Mostafa Modarres Mousavi, Narendran Annadurai, Sunčica Sukur, Faramarz Mehrnejad, Sajad Moradi, Lukáš Malina, Markéta Kolaříková, Vaclav Ranc, Ivo Frydrych, Roman Kouřil, Saman Hosseinkhani, Marián Hajdúch, Maryam Nikkhah

**Affiliations:** ^1^ Faculty of Medicine and Dentistry, Institute of Molecular and Translational Medicine Palacký University and University Hospital Olomouc Czech Republic; ^2^ Institute of Molecular and Translational Medicine, Czech Advanced Technologies and Research Institute Palacký University Olomouc Czech Republic; ^3^ Department of Nanobiotechnology, Faculty of Biological Sciences Tarbiat Modares University Tehran Iran; ^4^ Department of Nanobiotechnology and Biomimetics, School of Life Science Engineering University of Tehran Iran; ^5^ Nano Drug Delivery Research Centre, Health Technology Institute Kermanshah University of Medical Sciences Iran; ^6^ Department of Medical Biophysics, Faculty of Medicine and Dentistry Palacký University Olomouc Czech Republic; ^7^ Department of Biophysics, Faculty of Science Palacký University Olomouc Czech Republic; ^8^ Department of Biochemistry, Faculty of Biological Sciences Tarbiat Modares University Tehran Iran

**Keywords:** alpha‐synuclein, amyloid fibril, hydrophobic region, non‐amyloid component, Parkinson's disease, peptide inhibitor, synuclein seeding

## Abstract

Alpha‐synuclein (αSyn) is a 14‐kDa intrinsically disordered protein that aggregates into insoluble fibrils in synucleinopathies, including Lewy bodies, multiple system atrophy, and Parkinson's disease, contributing to neurotoxicity and disease progression. The ability of these fibrils to seed further aggregation of native protein is central to αSyn pathology. Here, we examined the broader non‐amyloid component (NAC) domain, focusing on how residues flanking the hydrophobic 68–71 (GAVV) motif of αSyn (residues 8–11 in NAC35) modulate nucleation, stability, and pathological seeding. Using full‐length NAC peptide and truncated variants, we show that the 68–71 (GAVV) stretch is critical for nucleation and aggregation into prion‐like fibrils. Peptide inhibitors targeting this hydrophobic region block the formation of seed‐competent fibrils. Molecular dynamics simulations showed that these inhibitors alter peptide–peptide interactions and contact key hydrophobic residues within the NAC domain. Further analysis indicates that residues beyond the 68–71 (GAVV) motif, such as 79–95, are critical for stabilizing fibrils and promoting seeding competency. Peptide B interactions with key hydrophobic motifs within the NAC domain were visualized *in silico*, offering mechanistic insights into how it disrupts aggregation.

## Graphical abstract

The hydrophobic 68GAVV71 stretch in the NAC domain of α‐synuclein is essential for nucleation, while residues 79–95 are required for fibril stability and seeding. Truncation or inhibition of 68GAVV71 prevents fibril formation, and truncation of 79–95 abolishes seeding competency, highlighting their distinct roles in α‐synuclein aggregation and pathology.

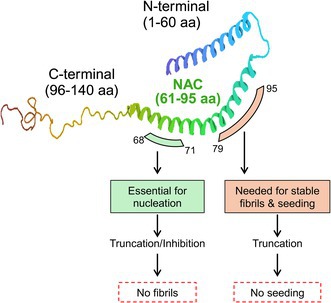

**Abbreviations**
AFM: atomic force microscopy
ANS: 8‐anilinonaphthalene‐1‐sulfonic acid
CFP: cyan fluorescent protein
MD: molecular dynamics
MSD: mean squared displacements
MST: microscale thermophoresis
NAC: non‐amyloid component
PB: Peptide B
PD: D‐amino acid peptide
PL: L‐amino acid peptide
PS: scrambled peptide
RMSD: root mean squared deviation
SASA: solvent‐accessible surface area
SPC/E: simple point charge extended
TEM: transmission electron microscopy
ThT: thioflavin T
UV‐Vis: ultraviolet‐visible
YFP: yellow fluorescent protein
αSyn: alpha‐synuclein

## Introduction

Amyloid fibrils of alpha‐synuclein (αSyn) are associated with dementia with Lewy bodies (DLB), multiple system atrophy, and Parkinson's disease [1, 2, 3]. These fibrils have been linked to neuronal death and neuroinflammation in cellular models [4, 5]. αSyn aggregates show prion‐like behavior, seeding further aggregation of native αSyn [6, 7]. Beyond neurological diseases, αSyn is associated with cancers, suggesting a potential overlap in disease mechanisms [8, 9]. Therefore, understanding the role of αSyn in one disease may help identify biomarkers and therapeutics for both diseases [10].

αSyn comprises an N‐terminal region (1–60 aa) that adopts an αhelix conformation when bound to membranes, a non‐amyloid component (NAC) region (61–95 aa) critical for aggregation, and a C‐terminal acidic tail (96–140 aa). The N‐terminal region harbors several mutation sites (A53T, A30P, and E46K) linked to familial Parkinson's disease [11]. The non‐NAC (P1 and P2) region is suggested to initiate αSyn aggregation by synergizing with the NAC and C‐terminal regions [12]. However, the NAC domain forms the core folded region of αSyn aggregates, playing a critical role in the aggregation process [13, 14, 15, 16]. Glu83 (E83) in the NAC acts as a negative regulator of amyloid formation, and mutations at this site, such as E83Q, accelerate aggregation and increase toxicity, as observed in DLB [2, 17].

Previous studies by Giasson *et al*. and Bodles *et al*. have emphasized the role of broader regions, such as 68–76 and 71–82, within the NAC domain in promoting αSyn aggregation [18, 19]. While these studies established the importance of hydrophobic stretch, they did not comprehensively address the specific contributions of flanking residues to aggregation dynamics and seeding competency. Our study extends these findings by systematically dissecting the broader NAC region and investigating inhibitors targeting critical residues, specifically, a previously validated peptide that mimics the 70–75 region of αSyn [20], which overlaps with the nucleation‐critical segment identified in our study. Understanding the role of specific residues is crucial, as even minor changes in this highly conserved sequence can profoundly influence aggregation dynamics and toxicity [17, 18]. Using full‐length hydrophobic NAC peptide and its truncated variants, we demonstrated the critical role of 68–71 (GAVV) residues in both the aggregation propensity and the seeding competency of the NAC region, findings not previously reported. Additionally, residues beyond this region, including residues 79–95, were shown to promote stable fibril formation and seeding activity. These findings highlight the complexity of αSyn aggregation and underscore the importance of considering the entire NAC region, not just the core hydrophobic stretch, in understanding αSyn aggregation.

## Results

### Truncation of residues 61–62 and 79–95 enhances NAC aggregation

**Fig. 1:**
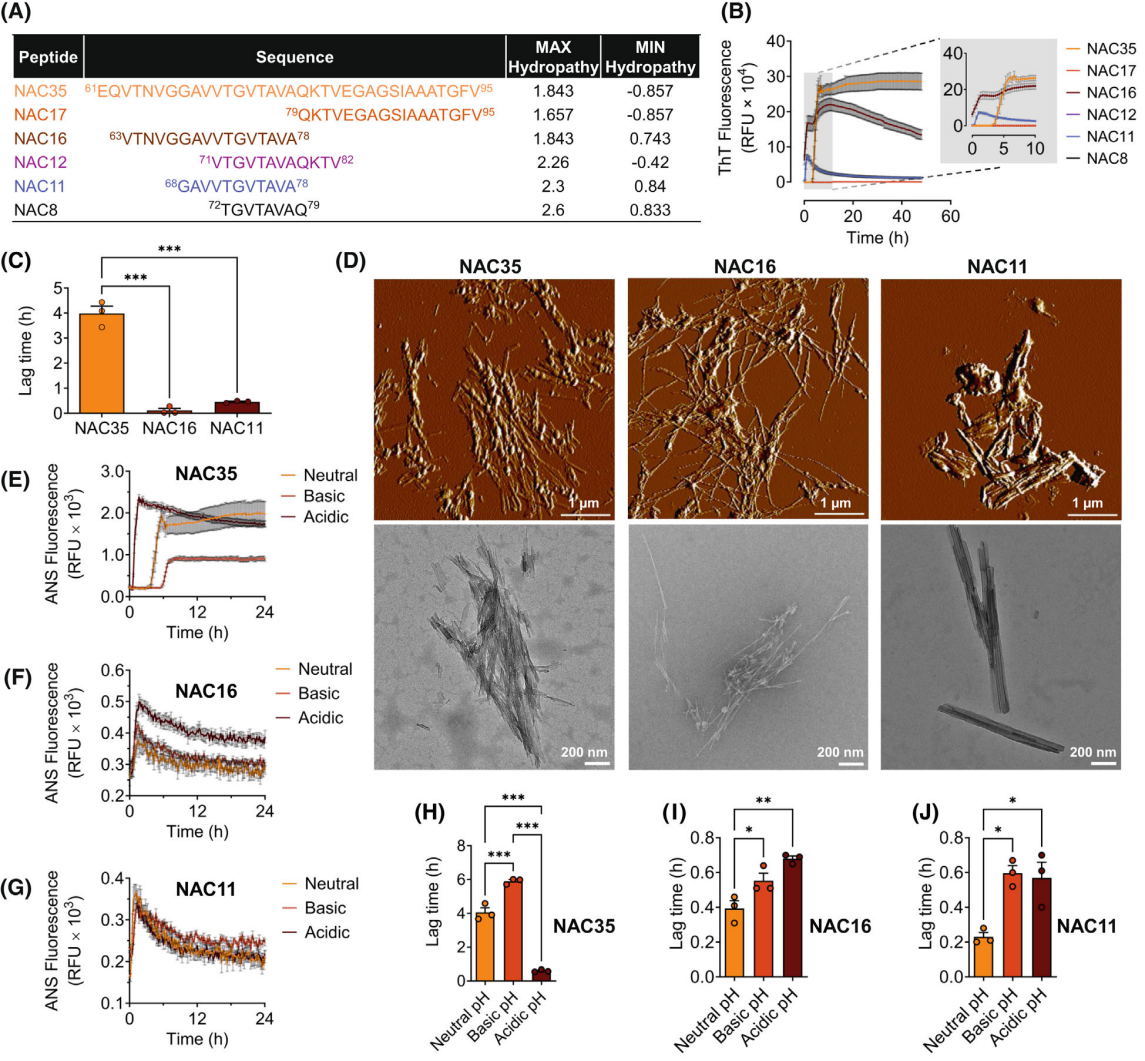
Truncation and pH modulate aggregation kinetics of NAC35, NAC16, and NAC11. (A) Amino acid sequences of the six NAC peptides used in the study. (B) Aggregation kinetics of NAC peptides over time, with the inset (gray box) showing the first 10 h of aggregation kinetics. (C) Lag time of NAC35, NAC16, and NAC11 aggregation, extracted from the first 10 h of data. (D) AFM (atomic force microscopy) and TEM (transmission electron microscopy) images of NAC aggregates after 48 h of aggregation. Scale bars: 1 μm (AFM) and 200 nm (TEM). (E–G) Aggregation kinetics of NAC peptides in neutral (pH 7.2), basic (pH 8.0), and acidic (pH 4.5) buffers. (H–J) Lag time of NAC35, NAC16, and NAC11 aggregation under neutral, basic, and acidic conditions. Mean ± SEM (*n* = 3 independent experiments), *P ≤ 0.05, **P ≤ 0.01, ***P ≤ 0.001 (One‐way ANOVA). *F* values and degrees of freedom are provided in the Supplementary Material. See Data Availability for access to source data (B) and (D–F).

Six distinct NAC peptides were generated by truncating specific regions of the NAC domain. NAC35 included the entire NAC domain, while NAC8 represented the shortest peptide, consisting of only eight residues (Fig. 1A). NAC11 (corresponding to αSyn 68–78), corresponding to the minimal toxic core described by Rodriguez *et al*. [16], was included to evaluate the role of this core in aggregation dynamics and fibril formation. Hydropathy analysis of these peptides was performed as described previously [21], revealing varying hydrophobic and hydrophilic characteristics across the sequences. NAC35 exhibited strong hydrophobicity at positions 8–12, whereas shorter peptides like NAC8 were predominantly hydrophilic (Fig. 1A).

The aggregation kinetics showed that NAC35 (corresponding to αSyn 61–95), NAC16 (corresponding to αSyn 63–78), and NAC11 (corresponding to αSyn 68–78) readily underwent aggregation, which correlates with their prominent hydrophobic peaks (1.84, 1.84, and 2.30, respectively; Fig. 1B). In contrast, NAC17 (corresponding to αSyn 79–95), NAC12 (corresponding to αSyn 71–82), and NAC8 (corresponding to αSyn 72–79), with lower hydrophobicity scores, did not aggregate. The presence of β‐sheet‐rich aggregates was also confirmed by fluorescence microscopy (Fig. S1). Notably, ThT curves of both NAC16 and NAC11 rapidly increased to a peak with a short plateau, followed by a decrease toward the starting point (Fig. 1B).

Next, we calculated the aggregation lag time using ThT readings between 0 and 10 h since this window corresponded to when NAC35, NAC16, and NAC11 aggregation curves reached their maximum peak. Truncated NAC16 and NAC11 peptides displayed a shorter lag time of aggregation than NAC35 (Fig. 1C).

Atomic force microscopy (AFM) and transmission electron microscopy (TEM) imaging revealed that NAC35 and NAC16 formed elongated fibrils with clumped tangles, whereas NAC11 aggregated into dense, sticky bundles lacking elongated tangles (Fig. 1D). In contrast, no fibrillar or aggregated structures were formed by NAC17, NAC12, and NAC8 after 48 h (Fig. S1), consistent with their inability to aggregate.

### pH sensitivity of NAC aggregation

An 8‐anilinonaphthalene‐1‐sulfonic acid (ANS) assay, which detects exposed hydrophobic regions [22], was performed to investigate how pH variations affect the aggregation behavior of NAC peptides. Altering pH did not induce NAC17, NAC12, and NAC8 aggregation. However, pH variations significantly influenced NAC35, NAC16, and NAC11 aggregation kinetics (Fig. 1E–G). For NAC35, acidic pH shortened the lag time while basic pH increased it. In contrast, NAC16 and NAC11 exhibited longer lag times under acidic and basic conditions than neutral pH (Fig. 1H–J).

### Solubility does not explain the lack of aggregation in NAC17, NAC12, and NAC8

**Fig. 2:**
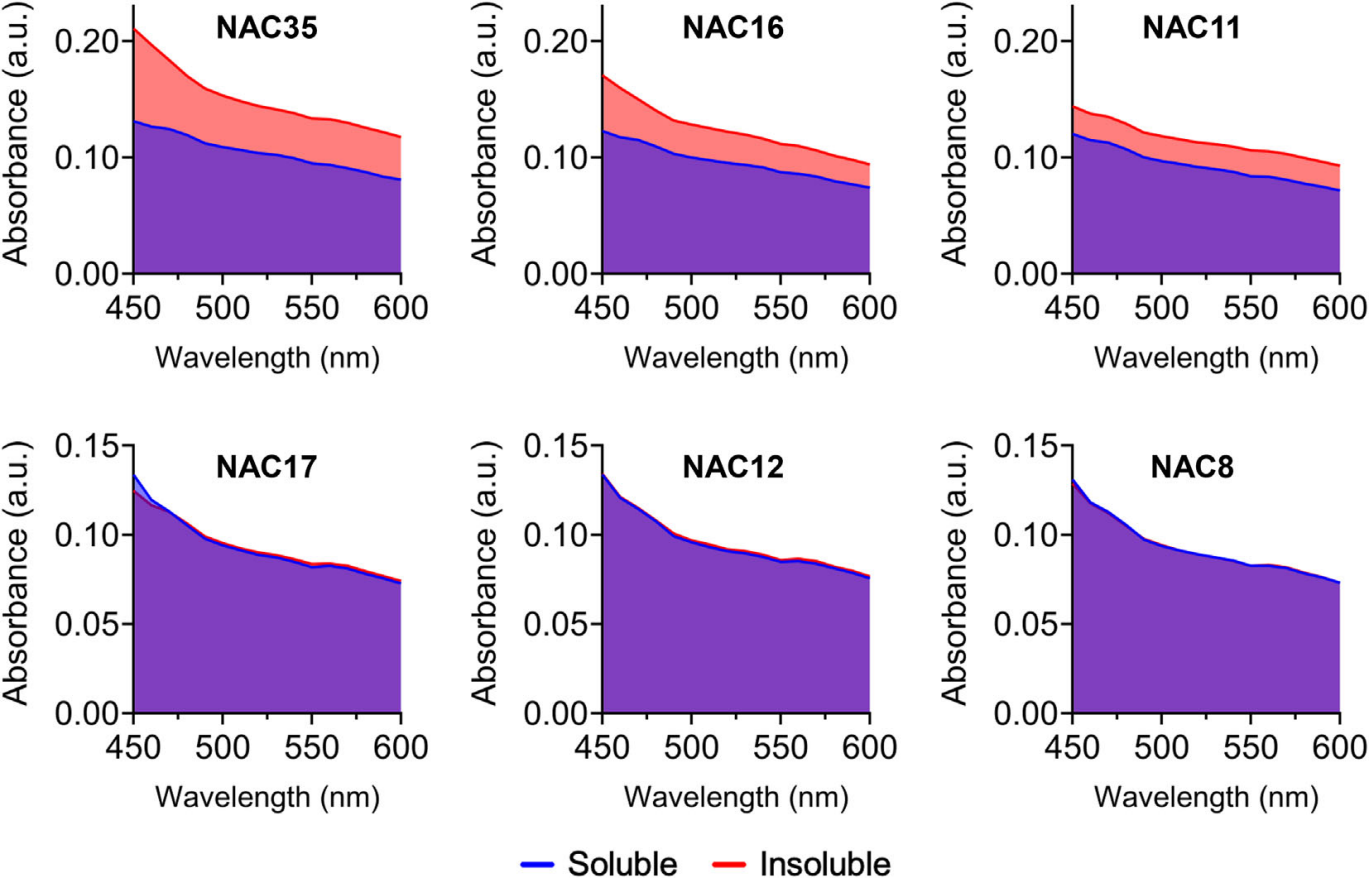
Solubility does not explain the lack of aggregation of NAC17, NAC12, and NAC8. UV–Vis absorption spectra of soluble and insoluble fractions of NAC peptides after 48 h of aggregation. Data represent the means of 3 replicates. See Data Availability for access to source data.

Hydropathy profiles indicated that NAC17, NAC12, and NAC8 were predominantly hydrophilic (Fig. 1A). UV–Vis spectrophotometry confirmed their solubility by showing indistinguishable spectra for soluble and insoluble fractions after incubation (Fig. 2). In contrast, aggregating peptides (NAC35, NAC16, NAC11) exhibited a clear increase in absorbance in the insoluble fraction, consistent with aggregate formation (Fig. 2; Fig. S2).

Structural changes of NAC35, NAC16, and NAC11 upon aggregation were further analyzed by Raman spectroscopy. The spectra of NAC35, NAC16, and NAC11 showed structural changes, marked by the increase in amide band intensities and new spectral signals (Fig. S3).

### NAC35 but not NAC16 and NAC11 preformed fibrils seed αSyn aggregation

**Fig. 3:**
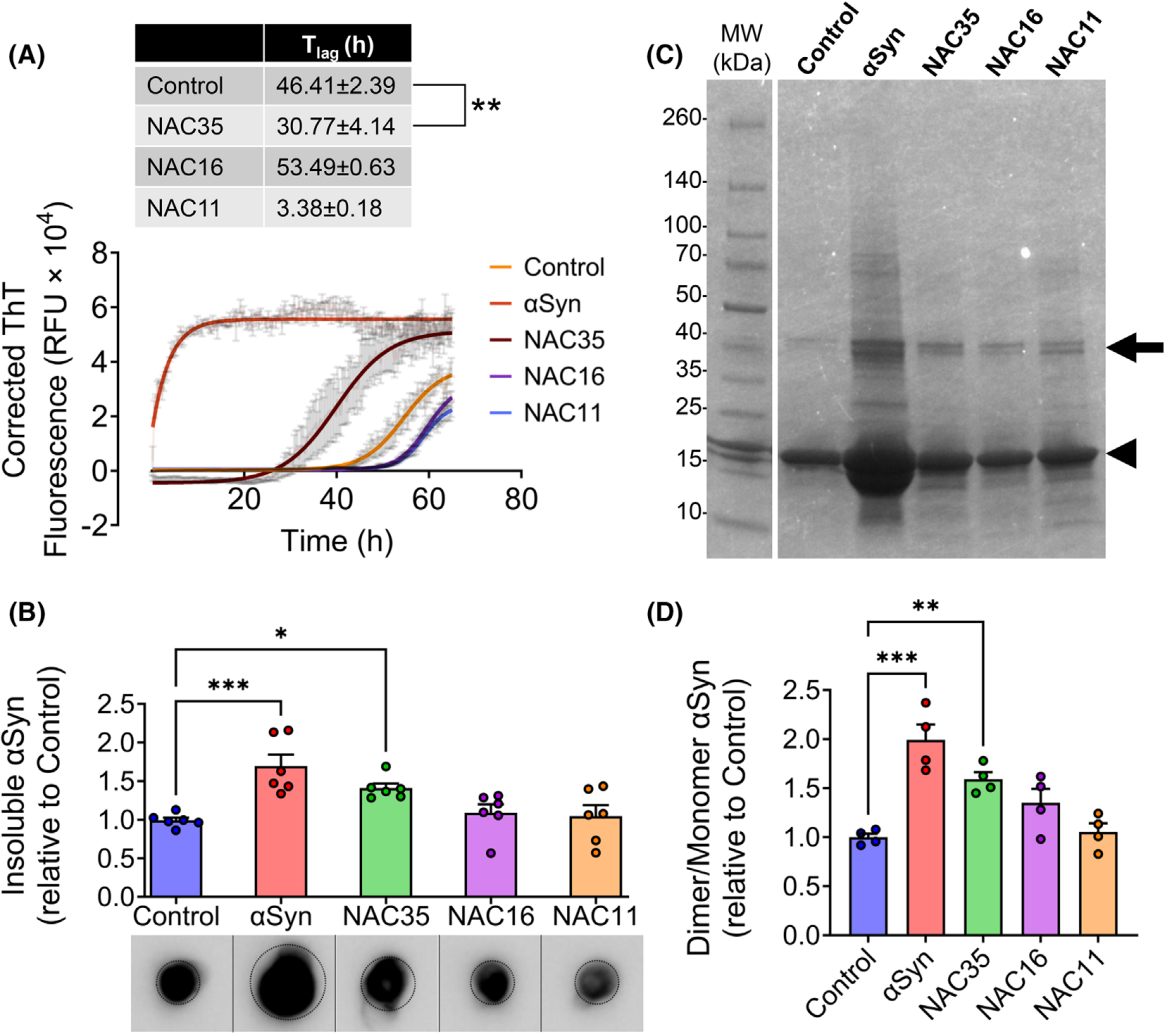
Preformed NAC35 fibrils seed monomeric αSyn aggregation. (A) Aggregation kinetics of monomeric αSyn seeded without (Control) or with preformed fibrils of αSyn, NAC35, NAC16, and NAC11 (4 : 1 monomer: seed ratio). Preformed αSyn fibrils were used as positive controls. Baseline correction was applied using stable ThT (thioflavin T) fluorescence values from preformed fibrils (Fig. S4) to ensure accurate quantification of seeded aggregation. The lag time for each condition is indicated. For αSyn seeded with preformed αSyn fibrils, the lag time was not calculated due to the absence of a detectable lag phase. (B) Quantification and dot blot analysis of insoluble fractions of αSyn aggregated for 72 h in the absence (control) or presence of preformed fibrils of αSyn, NAC35, NAC16, and NAC11. Dotted circles indicate the regions used for quantification. Vertical lines denote where the blot layout was adjusted to align sample lanes. The original, unmodified dot blot image is provided in Fig. S5. (C) Coomassie‐stained gel of insoluble fractions showing monomer (arrowhead) and dimer (arrow) bands. The full‐length gel image is shown in Fig. S5. (D) Quantification of the dimer‐to‐monomer band ratio showing increased αSyn dimerization in the presence of preformed αSyn and NAC35 fibrils. Mean ± SEM [*n* = 3 independent experiments for dot blot (B) and *n* = 2 for gel (D)], Control vs. Fibril‐seeded, *P ≤ 0.05, **P ≤ 0.01, ***P ≤ 0.001 (One‐way ANOVA). *F* values and degrees of freedom are provided in the Supplementary Material. See Data Availability for access to source data for (A).

Since Raman spectra of NAC35, NAC16, and NAC11 revealed amide band shifts, suggestive of β‐sheet changes [23], we examined whether their aggregates could act as seeds. Kinetics data showed that NAC35 fibrils, but not NAC16 and NAC11, significantly shortened the lag time of αSyn aggregation (Fig. 3A). Baseline correction was performed using stable ThT fluorescence values from preformed fibrils, as shown in Fig. S4. Although NAC16 and NAC11 form fibrils (Fig. 1D), they fail to seed αSyn aggregation, unlike NAC35 (Fig. 3A). αSyn seeded with preformed αSyn fibrils showed no detectable lag phase, indicating immediate aggregation, whereas unseeded αSyn aggregated with a longer lag time. Dot blot analysis confirmed the ThT results, revealing increased insoluble fractions in αSyn seeded with NAC35 fibrils (Fig. 3B). Quantifying dimer‐to‐monomer band ratios showed that preformed NAC35 fibrils, like αSyn fibrils, significantly increased αSyn dimerization (Fig. 3C,D). The full uncropped images of the dot blot and gel are shown in Fig. S5.

### Peptide inhibitors reduce dimerization and aggregation of αSyn

Building on our findings that the presence of residues 8–11 in NAC peptides, corresponding to αSyn 68–71, is necessary for efficient nucleation and aggregation, we investigated whether disrupting this hydrophobic region could inhibit full‐length αSyn aggregation. Truncating these residues abolished aggregation, and peptides containing them (e.g., NAC16, NAC11) aggregated rapidly (Fig. 1). To test whether targeting this site could suppress aggregation, we selected a previously validated inhibitor peptide based on the KISVRV sequence [20]. This segment mimics the 70–75 region of αSyn, a hydrophobic subregion overlapping with the 68–71 nucleation motif and implicated in fibril initiation [20]. This core sequence has been shown to inhibit αSyn fibril formation and dissolve preformed oligomers and was designed to engage the NAC domain through sequence and physicochemical complementarity [20].

**Fig. 4:**
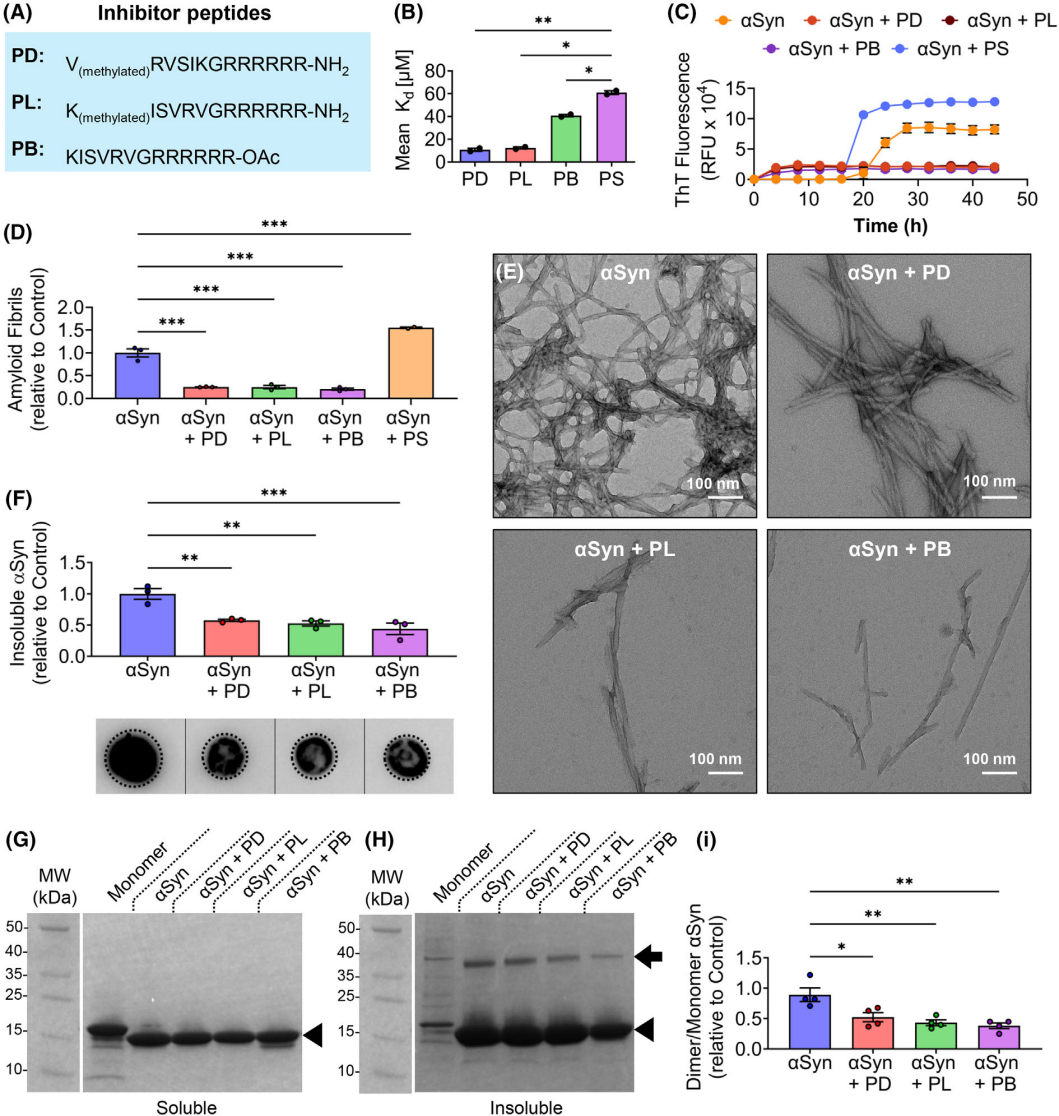
Inhibitor peptides targeting the hydrophobic region of αSyn reduce aggregation and modify fibril characteristics. (A) Sequences of PD (peptide D), PL (peptide L), and PB (peptide B) inhibitors. (B) Binding affinity (*K*_d_) of inhibitor peptides binding to αSyn. (C) Aggregation kinetics of αSyn in the absence or presence of PD, PL, PB, and PS (scrambled peptide) peptides. (D) Final ThT (thioflavin T) fluorescence readings quantifying amyloid fibrils formed at the end of the aggregation assay. (E) TEM (transmission electron microscopy) images of αSyn fibrils formed with or without PD, PL, or PB inhibitors. Scale bar: 100 nm. (F) Quantification and dot blot analysis of insoluble fractions of αSyn aggregated in the absence or presence of PD, PL, and PB. Dotted circles indicate the regions used for quantification. Vertical lines denote where the blot layout was adjusted to align sample lanes. The original, unmodified dot blot image is provided in Fig. S9. (G, H) Coomassie‐stained gel showing monomer (arrowheads) and dimer (arrow) bands in soluble (G) and insoluble (H) fractions for the indicated samples. Images of uncropped gels are shown in Fig. S10. (I) Dimer‐to‐monomer band ratio quantification in the insoluble fraction. Mean ± SEM [*n* = 3 independent replicates except for αSyn + PS in panel (C) where *n* = 2], *P ≤ 0.05, **P ≤ 0.01, ***P ≤ 0.001 (One‐way ANOVA). *F* values and degrees of freedom are provided in the Supplementary Material. See Data Availability for access to source data for (C).

In this study, we used an extended form of the previously validated KISVRV inhibitory sequence, adding a poly‐arginine tail (RRRRRR) to generate the full sequence KISVRVRRRRRR, which was common to all peptide variants tested. To enable future cellular applications, we synthesized three modified variants: peptide D (PD, composed of D‐amino acids), peptide L (PL, composed of L‐amino acids), and peptide B (PB, with an acetylated C terminus) (Fig. 4A). Potential effects related to stereochemistry, terminal modification, or the poly‐arginine tail extension were not evaluated in the present study. All inhibitor peptides contained the same KISVRV core sequence, previously shown to block αSyn fibrillation and disrupt preformed oligomers [20]. A scrambled control peptide (VSRKIVRRRRR), containing the same amino acid composition in a randomized sequence, was used to assess sequence specificity. This design follows the approach described previously [20], in which scrambled peptides (PS) preserve composition while eliminating motif‐specific interactions.

The affinity of PD, PL, PB, and a scrambled control peptide (PS) for αSyn was determined by microscale thermophoresis (MST). MST results showed a higher affinity of all inhibitor peptides for full‐length αSyn than the PS peptide (Fig. 4B; Fig. S6). While MST with full‐length αSyn successfully demonstrated the binding affinity of the inhibitors, attempts to use MST with truncated NAC peptides were not feasible due to technical challenges. Specifically, the small size and reduced structural stability of the NAC peptides hindered efficient fluorescent labeling (data not shown), which is essential for MST analysis and compromised the thermophoretic shifts required for reliable detection.

The effect of inhibitors on αSyn aggregation was evaluated by incubating αSyn with PD, PL, and PB at a 1 : 2 molar ratio and then monitoring aggregation by ThT assay. The kinetics data revealed that PD, PL, and PB are highly effective in inhibiting αSyn aggregation (Fig. 4C). To quantify the extent of inhibition, the final ThT reading for each treatment was normalized to that of the control group, providing a relative comparison of amyloid fibril formation. This analysis revealed that all inhibitors significantly reduced αSyn fibrils compared to the control (Fig. 4D). TEM analysis corroborated ThT results, showing reduced fibril density in the presence of inhibitors (Fig. 4E). The increase in αSyn aggregation in the presence of PS is likely due to its non‐specific binding (Fig. 4D), given that fibril morphology was unaffected (Fig. S7). PD, PL, PB, and PS peptides showed no signs of self‐aggregation (Fig. S8). Dot blot analysis and Coomassie staining revealed that inhibitors reduced the formation of insoluble fibrils and significantly decreased the dimer‐to‐monomer ratio of αSyn (Fig. 4F–I). The full uncropped images of the dot blot and gel are shown in Figs S9 and S10.

### αSyn fibrils formed in the presence of inhibitors have reduced seeding competency

Given that the inhibitors reduced αSyn dimerization, we next investigated the seeding competency of αSyn fibrils formed with inhibitors. To this end, we used HEK293T biosensor cells stably expressing αSyn (A53T) CFP/YFP‐tagged fusion proteins and quantified intracellular seeding using confocal microscopy and FRET flow cytometry, following the method described in the original study [24].

αSyn was incubated with PD, PL, and PB at a molar ratio of 1 : 2 for 72 h, after which the samples were collected and either used as a total fraction or separated into insoluble fractions by pelleting assay. To ensure consistency across all conditions, we measured the concentration of αSyn fibrils in each sample and used an equal concentration (1 μm) of total or insoluble fractions for cell transduction. This approach ensured that any observed differences in intracellular seeding are due to the structural and functional properties of the fibrils formed in the presence or absence of inhibitors rather than variations in the amount of αSyn fibrils applied.

**Fig. 5:**
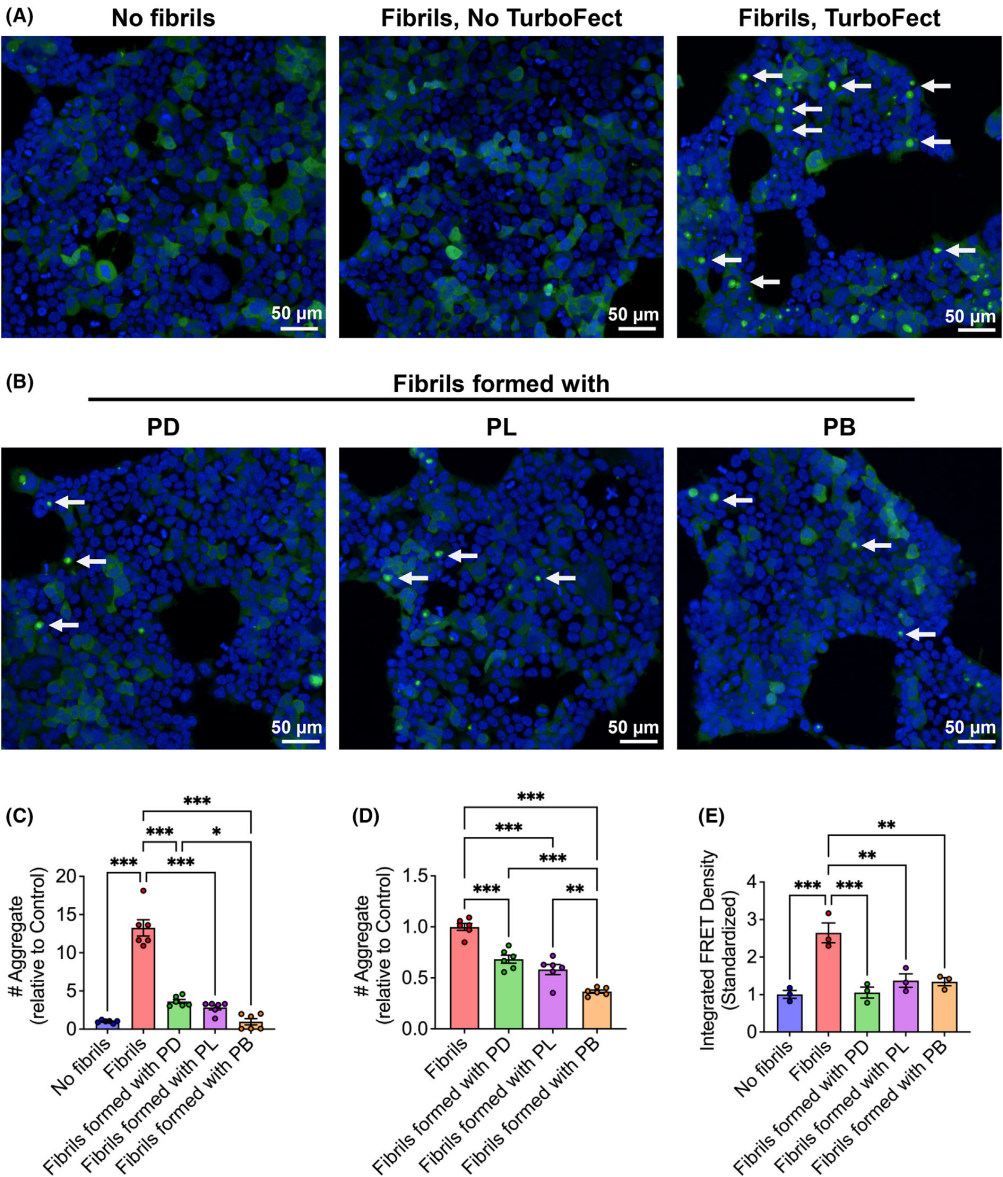
Inhibitor peptides prevent the formation of seed‐competent αSyn fibrils. (A, B) Confocal images of biosensor cells transduced with αSyn fibrils from the total fraction. CFP (cyan fluorescent protein)/YFP (yellow fluorescent protein) inclusions (white arrows) are absent without fibril transduction but appear with fibrils in the presence of TurboFect. Cells treated with PD (peptide D), PL (peptide L), or PB (peptide B) fibrils show reduced inclusions. Hoechst‐33342 staining is blue. Scale bar: 50 μm. (C, D) Number of CFP/YFP inclusions in cells transduced with αSyn fibrils from the total fraction (C) or insoluble fraction (D). (E) Standardized integrated FRET (fluorescence resonance energy transfer) density of cells transduced with αSyn fibrils formed with or without inhibitors from the total fraction. Scatter plots showing the gating of cells are shown in Fig. S11. Mean ± SEM (*n* = 3 independent experiments), *P ≤ 0.05, **P ≤ 0.01, ***P ≤ 0.001 (one‐way ANOVA). *F* values and degrees of freedom are provided in the Supplementary Material. See Data Availability for access to source data for (C–E).

Fluorescent CFP/YFP inclusions were not observed in the absence of exogenous fibrils (Fig. 5A) or when cells were transduced with fibrils without the TurboFect transfection reagent (Fig. S11), consistent with previous reports that liposome‐mediated transduction facilitates efficient seeding in these biosensor cells [24]; though passive uptake of sonicated fibrils has also been shown to induce seeding [25]. Cells transduced with fibrils formed in the presence of inhibitor peptides exhibited significantly fewer CFP/YFP inclusions than those transduced with control total or insoluble αSyn fibrils (Fig. 5B–D). PB was the most effective in reducing the seed competency of αSyn fibrils compared to the other inhibitors (Fig. 5C,D). The inhibitor peptides alone did not induce seeding, as cells transduced with only the inhibitors showed no CFP/YFP inclusions (Fig. S11). FRET cytometry data further corroborated our CFP/YFP inclusion count results (Fig. 5E), confirming the reliability of inclusion counting as demonstrated in this study [26].

### Sequence‐specific modulation of NAC peptide aggregation by inhibitor peptides

**Fig. 6:**
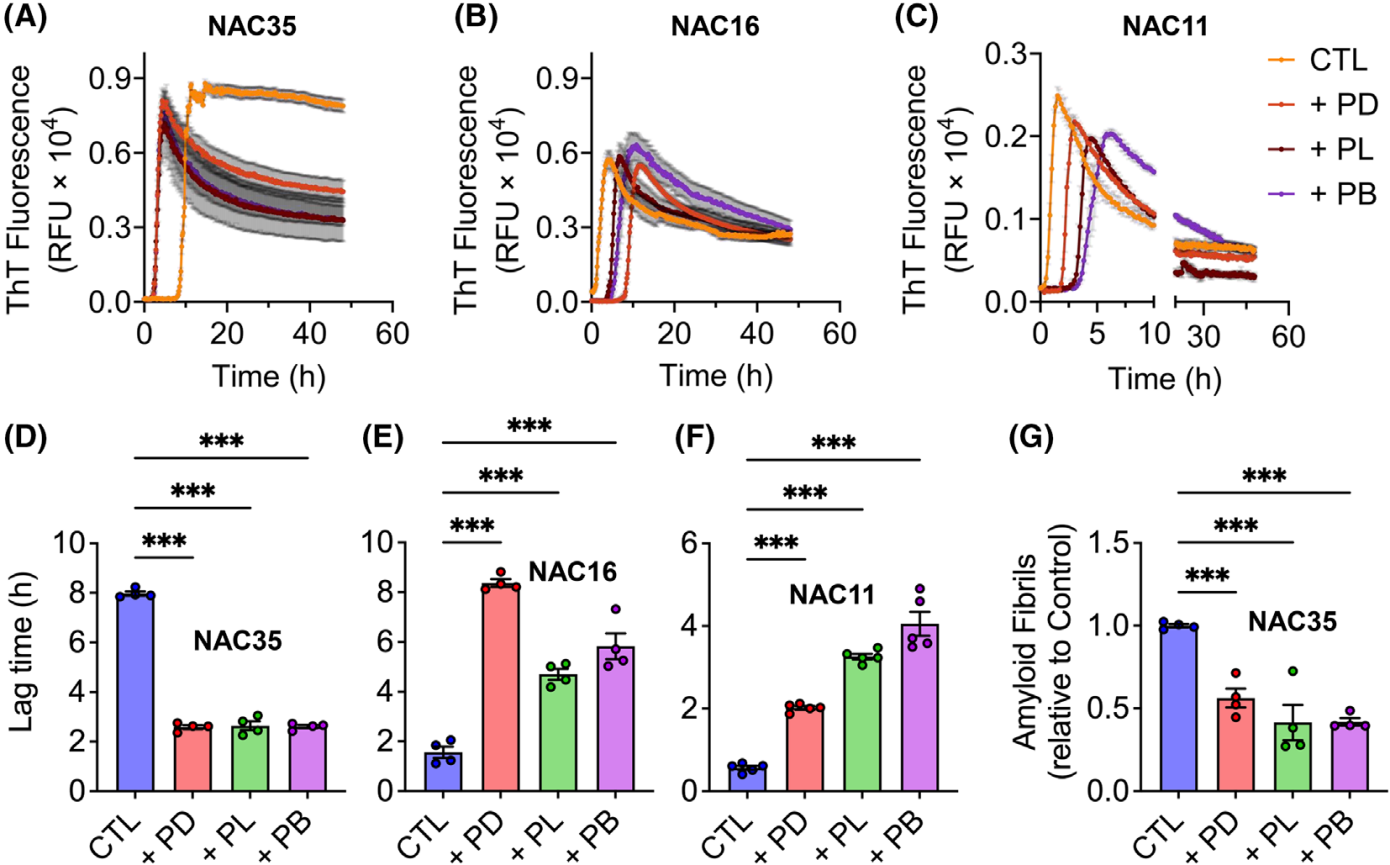
Inhibitor peptides modulate NAC aggregation kinetics and lag times. (A–C) Aggregation kinetics of NAC35, NAC16, and NAC11 without (CTL) or with PD (peptide D), PL (peptide L), and PB (peptide B) inhibitors. (D–F) Lag time of aggregation in the absence (CTL) or presence of inhibitor peptides. (G) Final ThT fluorescence readings quantifying amyloid fibril formation at the end of the aggregation assay. Mean ± SEM (*n* = 4 independent experiments), ***P ≤ 0.001 (One‐way ANOVA). *F* values and degrees of freedom are provided in the Supplementary Material. See Data Availability for access to source data for (A–C).

To further investigate the region‐specific effects of inhibitor peptides on aggregation, we studied their interactions with NAC peptides. By focusing on NAC35, NAC16, and NAC11, we aimed to identify aggregation‐prone stretches within the NAC domain. NAC16 (63–78) and NAC11 (68–78) aggregated efficiently, whereas peptides lacking these residues did not aggregate (Fig. 1). NAC peptides were incubated with inhibitor peptides at a 1 : 2 molar ratio, and aggregation was monitored by ThT assay in the neutral buffer. Control experiments demonstrated that inhibitor peptides showed no signs of self‐aggregation (Fig. S7). The results revealed distinct inhibitory effects on NAC peptide aggregation: inhibitors shortened the lag time of NAC35 aggregation (Fig. 6A,D), while significantly increasing the lag time of NAC16 and NAC11 aggregation (Fig. 6B,C,E,F). Despite the reduced lag time of NAC35 aggregation, the total amount of amyloid fibrils formed was markedly lower in the presence of inhibitors (Fig. 6G).

**Fig. 7:**
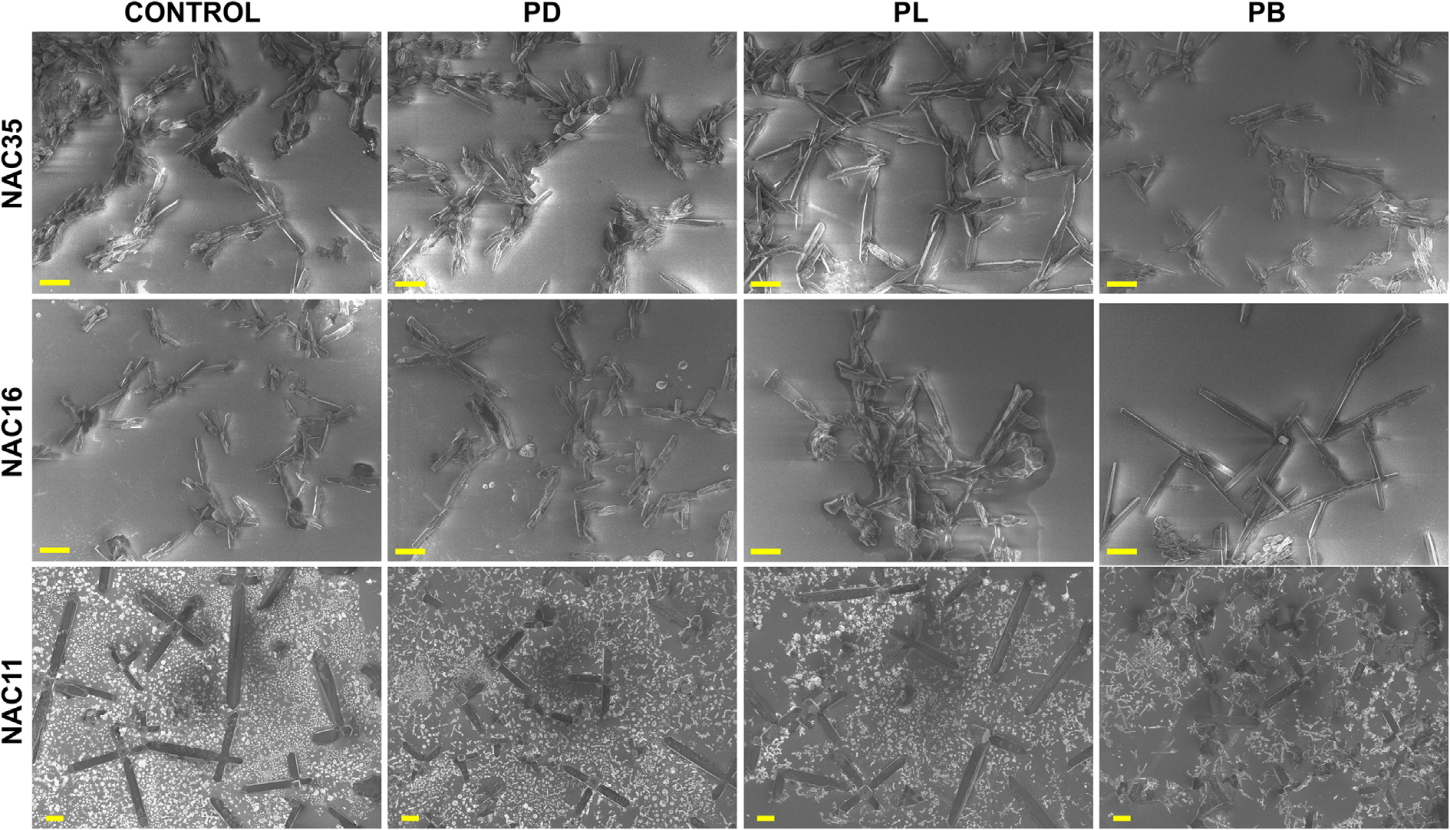
Fibril fragmentation is visible in NAC peptide samples treated with inhibitors. SEM (scanning electron microscopy) images of NAC35, NAC16, and NAC11 fibrils formed in the absence (control) and presence of PD (peptide D), PL (peptide L), and PB (peptide B) inhibitors. Scale bar: 10 μm (NAC35 and NAC16) and 50 μm (NAC11). Higher‐magnification NAC11 images are provided in Fig. S12.

This observation was corroborated by scanning electron microscopy (SEM) analysis (Fig. 7). In the absence of inhibitors, NAC35 formed dense networks of long, bundled fibrils. Inhibitor‐treated NAC35, particularly with PB, contained visibly shorter fibrils that were less bundled and appeared fragmented. NAC16 formed continuous fibrils under control conditions, while PD, PL, and PB treatments resulted in shorter, discontinuous fibrillar structures. NAC11 formed large, clustered fibrils that extended beyond the field of view at the magnification used for NAC35 and NAC16. To allow comparison, NAC11 samples were imaged at a lower magnification (Fig. S12). In the presence of inhibitors, NAC11 showed reduced fibril continuity and more dispersed fragments at both magnifications (Fig. 7; Fig. S12). No distinct morphologies or additional structures were observed that would indicate heterogeneous co‐aggregation between inhibitors and NAC peptides. The inhibitors alone did not form fibrillar or amorphous aggregates (Fig. S13).

### PB inhibitor disrupts NAC aggregation dynamics through structural instability

**Fig. 8:**
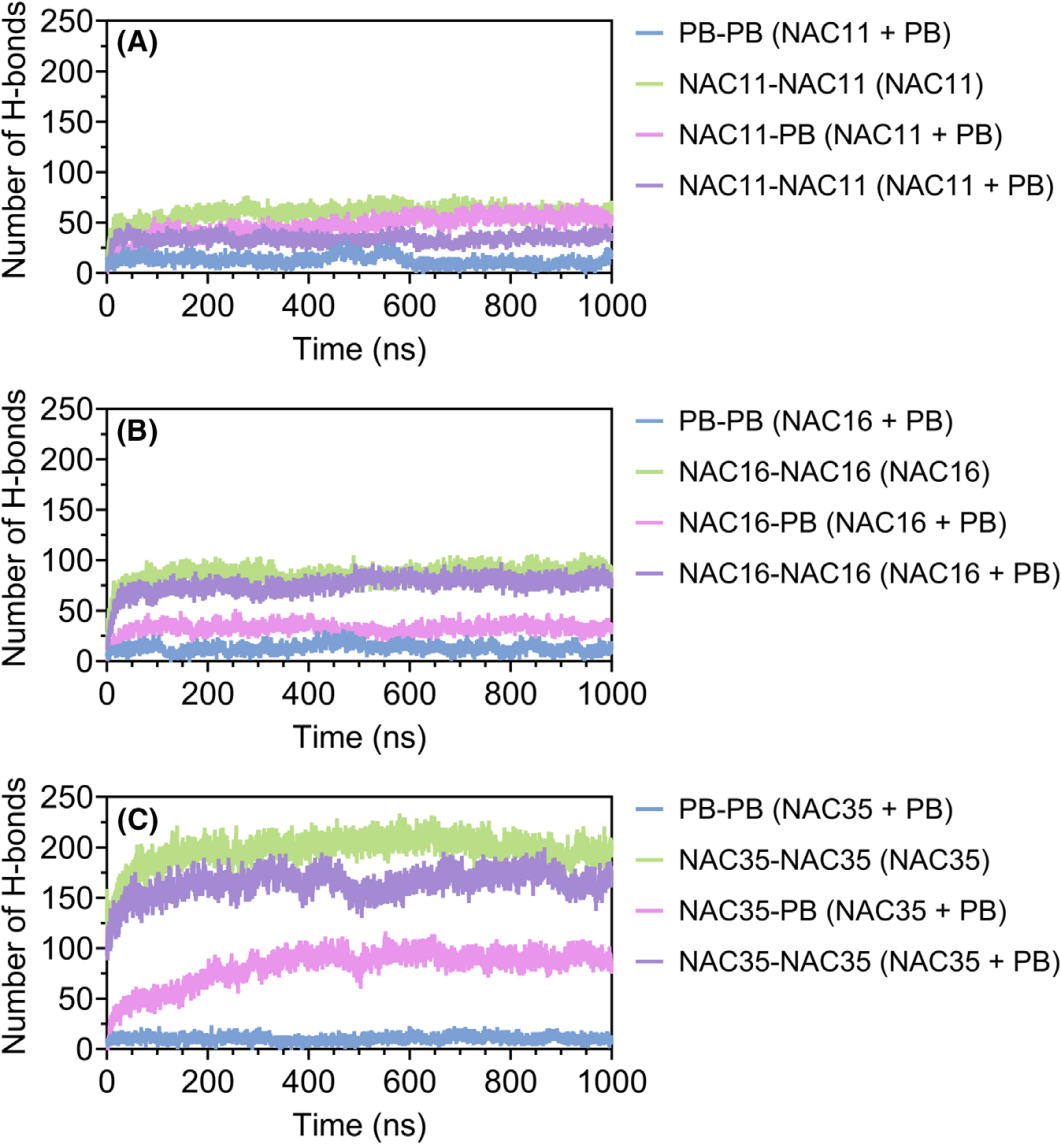
Hydrogen bond distribution in NAC and NAC + PB systems. The number of hydrogen bonds is shown for each simulated system: NAC‐only (NAC) and NAC in the presence of PB (peptide B) inhibitor (NAC + PB). NAC–NAC refers to hydrogen bonds between NAC peptides, NAC–PB to hydrogen bonds between NAC and PB, and PB–PB to hydrogen bonds between PB peptides. Single‐trajectory simulation; representative results are shown. See Data Availability for access to source data.

Molecular dynamics (MD) simulations were performed to assess how PB interacts with NAC peptides and alters their structural behavior. In the absence of PB, NAC peptides exhibited extensive intermolecular hydrogen bonding (Fig. 8). However, in systems containing PB, the number of NAC–NAC hydrogen bonds decreased, while NAC–PB hydrogen bonding increased.

**Fig. 9:**
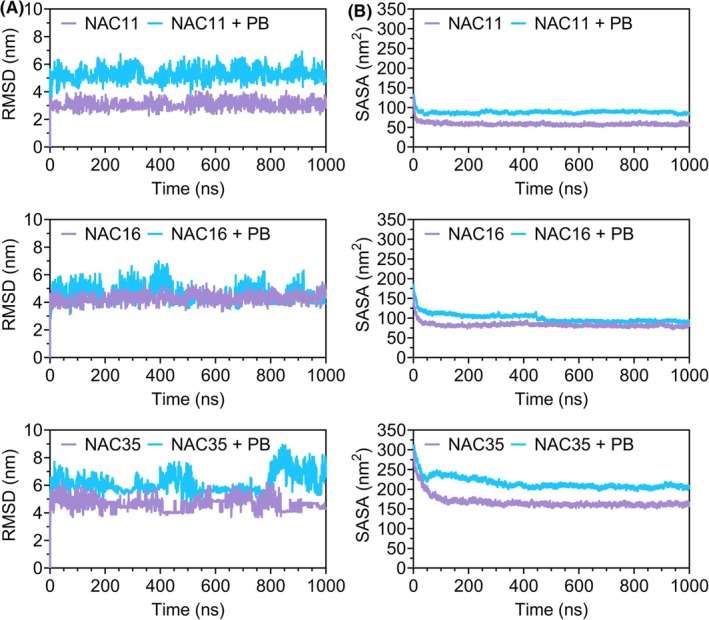
RMSD and SASA profiles of NAC peptides in the presence and absence of PB. (A) Root mean square deviation (RMSD) over the 1000 ns molecular dynamics simulation for NAC‐only and NAC + PB (peptide B) systems. (B) Solvent‐accessible surface area (SASA) measurements for NAC peptides in the same systems. Single‐trajectory simulation; representative results shown. See Data Availability for access to source data.

The root mean square deviation (RMSD) values of the Cα atoms showed consistently higher values in NAC + PB systems compared to NAC‐only systems (Fig. 9A). This pattern was observed across all three NAC peptides, with NAC11 + PB exhibiting the highest RMSD. Solvent‐accessible surface area (SASA) values were also higher in NAC + PB systems than in NAC‐only systems (Fig. 9B).

**Fig. 10:**
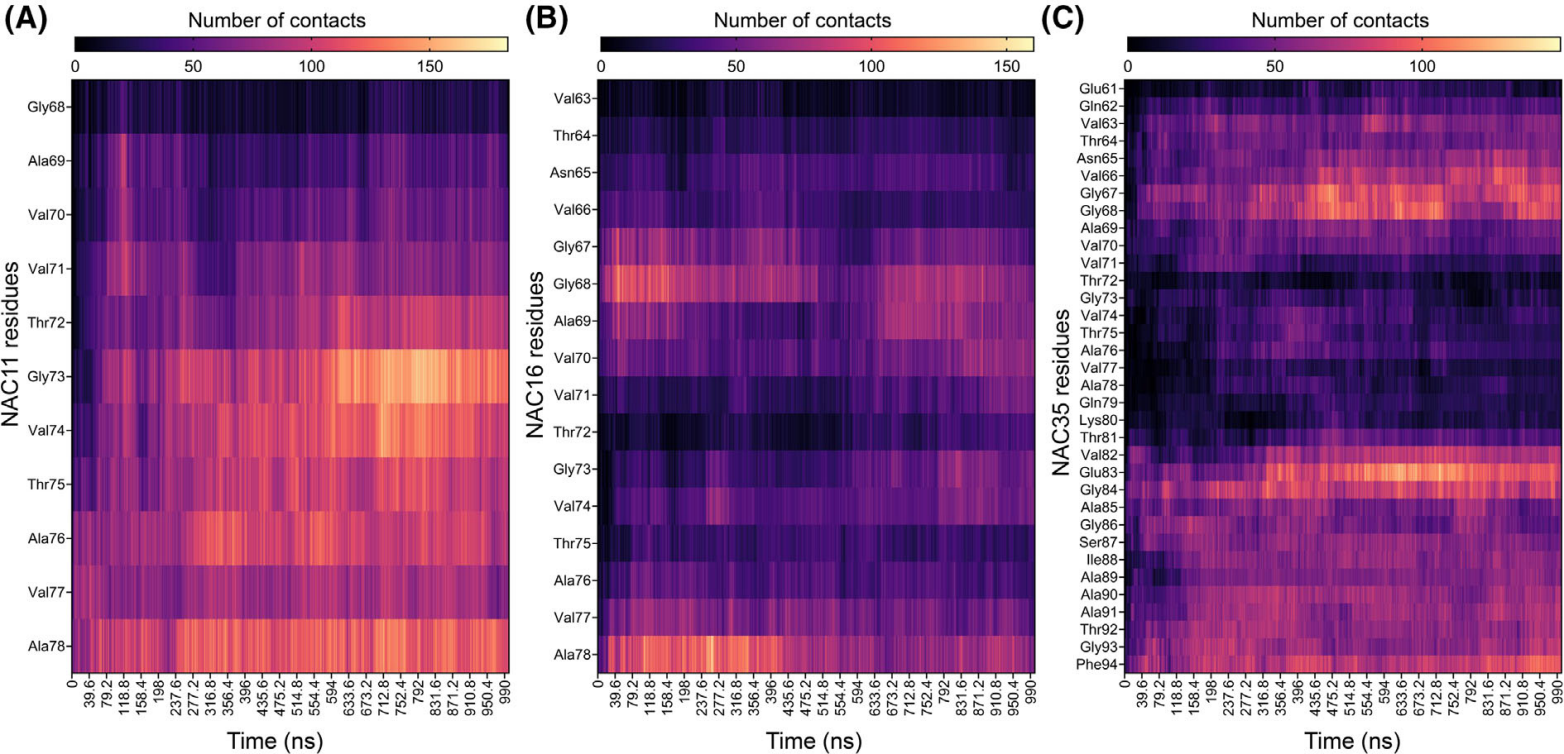
Residue‐level contact analysis between NAC peptides and PB. Heatmaps showing the number of contacts between individual NAC residues and PB (peptide B) in three systems: (A) NAC11 + PB, (B) NAC16 + PB, and (C) NAC35 + PB. Single‐trajectory simulation; representative results shown. See Data Availability for access to source data.

To assess residue‐specific interactions between NAC peptides and the PB inhibitor, we quantified the number of contacts formed by each NAC residue with PB throughout the simulation (Fig. 10). The contact heatmaps revealed that NAC11 formed the most extensive and persistent interactions with PB, particularly across residues Ala69 to Ala78 (Fig. 10A). In NAC16, PB primarily contacted two regions: Gly67 to Val70 and Val77 to Ala78 (Fig. 10B). For NAC35, PB interactions were more dispersed, with notable contact frequencies observed in two regions: Val66 to Ala69 and Glu83 to Ala85 (Fig. 10C).

**Fig. 11:**
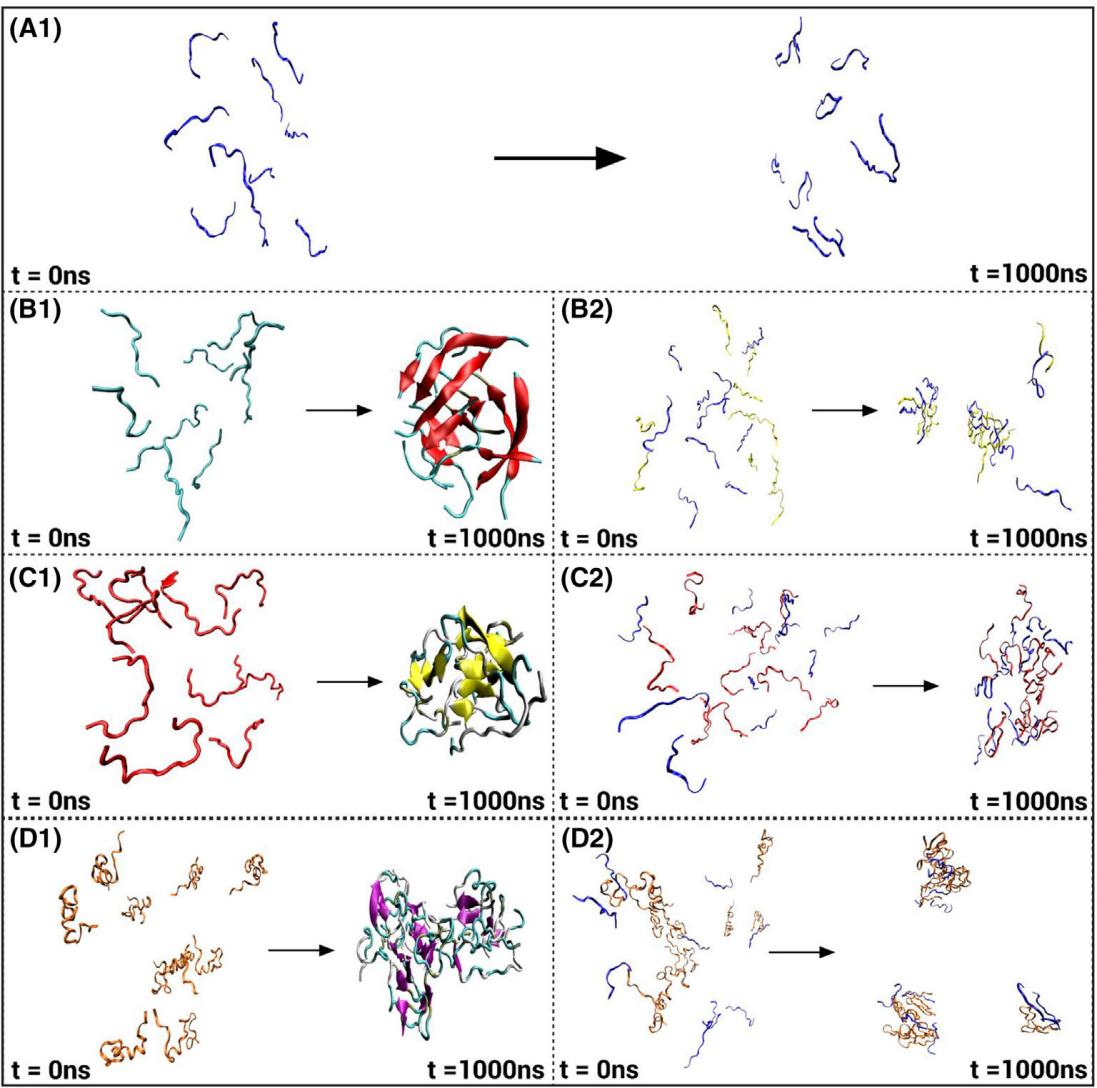
Structural evolution of NAC peptides in the presence and absence of PB inhibitor. Representative molecular frames at T = 0 and T = 1000 ns are shown for: (A) PB (peptide B; purple), (B1) NAC11 (cyan) and (B2) NAC11 with PB (purple), (C1) NAC16 (red) and (C2) NAC16 with PB (purple), (D1) NAC35 (orange) and (D2) NAC35 with PB (purple). Prepared using vmd (Visual Molecular Dynamics) software.

Representative molecular frames extracted at T = 0 and T = 1000 ns show that PB remained structurally stable and maintained consistent interactions with NAC peptides over the stimulation period (Fig. 11). In the absence of PB, NAC peptides appeared visually clustered into compact groupings by the end of the 1 μs simulation. In contrast, in the presence of PB, the peptides remained spatially separated and extended, with no evident clustering.

## Discussion

Prion‐like propagation of αSyn aggregation is a critical pathological process in synucleinopathies, with the NAC domain (61–95) forming the core of aggregates [13, 14, 15, 16]. This study addresses gaps in understanding how residues in and around the NAC contribute to aggregation, fibril stability, and prion‐like behavior. By systematically testing truncated NAC peptides, we reveal distinct contributions of residues within and flanking the 71–82 core sequence to aggregation dynamics, nucleation, fibril stability, and seeding competency.

Previous studies have identified key sequences within the NAC domain, including the 71–82 region, the 68–76 stretch, and the toxic NACore fragment [16, 18, 19]. While these studies established the role of the 68–82 region in aggregation, they did not explore how residues flanking the core region contribute to nucleation efficiency, fibril stability, and prion‐like behavior. In contrast, our study systematically dissected the NAC region, pinpointing the unique contributions of residues 68–71 and their interactions with adjacent sequences.

We provide evidence that truncating 68–71 (GAVV) abolishes aggregation, as NAC12 and NAC8, which lack these residues, do not aggregate, highlighting their essential role in nucleation. While the 71–88 region promotes aggregation in NAC35 (61–95), it is insufficient alone, as demonstrated by the lack of aggregation in NAC12 (71–82). In contrast, peptides such as NAC16 (63–78) and NAC11 (68–78), which include flanking residues, aggregate efficiently, underscoring the critical contributions of regions outside the core stretch. Our results clarify that aggregation involves a broader interplay of residues, demonstrating that flanking residues stabilize fibril structures and enhance aggregation kinetics.

This interpretation is supported by Raman spectroscopy, which revealed band shifts and increased intensities in NAC35, NAC16, and NAC11 ‐ changes characteristic of β‐sheet formation and consistent with those observed in αSyn fibrils [23]. In contrast, peptides such as NAC17, NAC12, and NAC8 failed to aggregate under the same conditions. This lack of aggregation appears to result from insufficient hydrophobic interactions rather than poor solubility, as these peptides remained fully soluble throughout the assays.

While previous reports and our hydropathy analysis identified multiple hydrophobic segments across the NAC region, our focus was specifically on αSyn residues 68–71 (corresponding to NAC residues 8–11). Although residue 72 is threonine and polar, we observed that truncating just beyond Val71 abolishes aggregation entirely, highlighting 68–71 (GAVV) as likely the minimal nucleation motif. Other hydrophobic stretches, such as residues 88–91, were not explored in this study because they did not appear within the aggregation‐prone motifs identified by prior structural studies and our experimental truncations. Moreover, residues 68–71 displayed distinct responsiveness to pH variation in our aggregation assays, making them a particularly suitable and experimentally tractable focal point.

We show that residues outside the core hydrophobic stretch (68–78) influence aggregation behavior under different pH conditions. This includes hydrophobic residues such as 8–11 in NAC35 (corresponding to αSyn 68–71), 6–8 in NAC16 (corresponding to αSyn 68–70), and 4 and 7 in NAC11 (corresponding to αSyn 71 and 74), which may contribute to the observed pH‐dependent changes in aggregation lag time. Waxman *et al*. identified hydrophobic stretches, including residues 71–82 and 74–79, in αSyn amyloid formation [17]. Our findings extend this by demonstrating that residues beyond the core 71–82 stretch, such as those in NAC35, contribute significantly to aggregation stability and dynamics, particularly under acidic conditions. The pH‐dependent effects of NAC35 aggregation likely result from the protonation or deprotonation of ionizable residues, modulating hydrophobic and electrostatic interactions critical for aggregation. Our observation of NAC35 aggregation in an acidic buffer aligns with the mechanism described by Hoyer *et al*. [22], where lower pH conditions reduce electrostatic repulsion among acidic residues such as Asp and Glu due to increased protonation, thereby promoting aggregation. We also observed that aggregating NAC35 in a basic buffer (pH 8.0) increased the aggregation lag time. Although not directly examined previously, we speculate that this effect may be explained by destabilized hydrophobic interactions involving residues such as Val66, Val70, and Val74 [18, 19], and by enhanced electrostatic repulsion, presumably arising from the deprotonation of Glu83 under basic conditions [17].

Aggregation kinetics, as reflected in lag time (nucleation efficiency) and the endpoint fibril amount, indicating fibril stability, further demonstrate the functional roles of NAC peptides. While NAC16 and NAC11 exhibit faster nucleation than NAC35, this difference is not solely due to the presence of 68–71 (GAVV), as NAC35 also contains this hydrophobic stretch. Previous MD studies have reported that NACore (NAC11) forms stable antiparallel β‐sheet dimers with a favorable binding free energy [27, 28, 29], identifying it as a critical amyloidogenic sequence. Our experimental results, showing that NAC11 aggregates efficiently and forms dense fibrillar bundles, extend these computational findings, suggesting that secondary interactions beyond dimerization contribute to its aggregation. Instead, NAC35 includes additional N‐ and C‐terminal flanking residues (61–67 and 79–95, respectively) relative to the NAC11 core, which may stabilize transient interactions and reduce hydrophobic exposure, modulating nucleation dynamics.

Notably, the ThT fluorescence of NAC11 and NAC16 rose quickly before declining, a pattern consistent with aggregate precipitation. This atypical behavior may reflect the formation of large aggregates that precipitate and sequester monomers, reducing their concentration, as noted by others for Aβ aggregation [30]. However, the fibrils formed by NAC16 and NAC11 are less stable and lack the structural integrity required for seeding. While 68–71 contributes to nucleation efficiency, the absence of stabilizing residues (79–95), which are present in NAC35 but lacking in NAC16 and NAC11, may prevent them from maintaining the fibril architecture necessary for effective templating and seeding [31, 32]. Conversely, NAC35, which includes these stabilizing residues, shows slower nucleation but forms fibrils with enhanced stability and robust seeding competency.

The increase in the αSyn dimer‐to‐monomer ratio observed in the presence of preformed NAC35 fibrils indicates that these fibrils enhance aggregation‐prone interactions, aligning with their observed seeding activity. The detection of SDS‐resistant dimers may reflect the formation of stable β‐sheet‐mediated intermediates, as supported by MD simulations showing that NACore peptides form thermodynamically favored antiparallel β‐sheet dimers that resist dissociation even under aqueous conditions [29]. In contrast, NAC16 and NAC11 fibrils did not promote dimerization or aggregation, further confirming their lack of seeding competency. This dual analysis of lag time and endpoint fibril formation reveals the interplay between residues critical for nucleation and those promoting stability [31, 32]. It further explains how specific segments within the NAC region may collectively drive αSyn aggregation and its pathological behavior. Table 1 summarizes the key findings on truncated NAC peptides and their contributions to nucleation dynamics, fibril stability, seeding competency, and responses to inhibitors.

**Table 1:** Summary of aggregation properties and inhibitor effects of NAC peptides. Key findings on nucleation dynamics, fibril stability, seeding competency, and responses to inhibitors for truncated NAC peptides. The results emphasize the roles of core and flanking residues in aggregation and provide comparisons with previous studies.

Peptide	NAC residues	αSyn residues	Key observations	Inhibitor effect	Comparison with prior findings
NAC35	1–35	61–95	Slow nucleation; forms robust fibrils with enhanced stability and effective seeding of αSyn aggregation	Disrupts nucleation and reduces fibril formation	Extends Bodles *et al*. [18]: Highlights the role of 79–95 in enhancing fibril stability and seeding beyond core regions (68–76)
NAC17	19–35	79–95	No nucleation or fibril formation observed.	Not applicable (no aggregation observed)	Shows that 79–95 alone does not aggregate but contributes to stability when paired with NACore residues (68–78)
NAC16	3–18	63–78	Rapid nucleation; moderately stable fibrils; lacks seeding ability	Delays nucleation and reduces fibril formation	Builds on Bodles *et al*. [18]: Demonstrates that flanking residues (63–67, 72–78) enhance aggregation but are insufficient for fibril stability or seeding
NAC12	11–22	71–82	No nucleation or fibril formation observed	Not applicable (no aggregation observed)	Contradicts Giasson *et al*. [19]: Shows 71–82 alone cannot form fibrils, indicating flanking residues are required for aggregation
NAC11	8–18	68–78	Rapid nucleation; forms unstable fibrils prone to precipitation; lacks seeding ability	Disrupts nucleation and reduces fibril formation	Builds on Rodríguez *et al*. [16]: Confirms NACore (68–78) as essential for nucleation but insufficient for fibril stability or seeding
NAC8	12–19	72–79	No nucleation or fibril formation observed	Not applicable (no aggregation observed)	Extends Bodles *et al*. [18]: Confirms residues 72–79 alone cannot aggregate, emphasizing the indispensability of 68–71 for aggregation

While multiple hydrophobic segments exist within the NAC domain, including hydrophobic or particularly hydrophobic stretches such as residues 88–91 and 76–78, our study focused on residues 68–71 due to their demonstrated role in nucleation efficiency and fibril formation. The elimination of aggregation upon truncation of this region suggests it serves as a critical determinant of aggregation dynamics. Furthermore, its central positioning within the NAC domain aligns with previously identified aggregation‐prone motifs, reinforcing its relevance as a therapeutic target. Peptide inhibitors targeting the hydrophobic NAC region further elucidated the role of specific NAC residues in seeding competency. All inhibitors effectively blocked recombinant αSyn aggregation and reduced insoluble fibril formation. The observed reduction in SDS‐resistant αSyn dimers in the presence of inhibitors may reflect an alteration in early aggregation behavior, although the specific structural intermediates remain unclear. Given that the prion‐like behavior of αSyn fibrils relies on their structural features [33, 34, 35], the loss of seeding competency in fibrils formed in the presence of inhibitors underscores the importance of these structural disruptions.

The distinct responses of NAC16 and NAC11, which have fewer hydrophobic residues than NAC35, are consistent with the idea that inhibitors act on nucleation‐relevant segments within the NAC domain, such as the 68–71 (GAVV) motif or nearby hydrophobic residues [17]. The significant delay in aggregating these truncated NAC peptides in the presence of inhibitors indicates the disruption of the hydrophobic interaction needed for nucleation. In contrast, NAC35 showed a shortened lag time in the presence of inhibitors.

The acceleration of aggregation likely arises from the transient stabilization of early aggregation intermediates involving hydrophobic residues such as 68–71. While inhibitors appear to disrupt hydrophobic contacts across all NAC variants, the longer NAC35 sequence may enable partial self‐association prior to full inhibitor engagement. This biphasic behavior may reflect an initial phase of partial aggregation facilitated by NAC35's extended sequence, followed by inhibitor‐mediated disruption of fibril integrity.

This interpretation is supported by SEM images, which show that fibrils formed by NAC35 in the presence of inhibitors, particularly PB, are shorter and more fragmented than in the control condition, consistent with the reduced endpoint ThT signals. These morphological effects were not limited to NAC35; NAC16 and NAC11 also showed disrupted fibril architecture under inhibitor treatment. Although their endpoint ThT signals did not decrease substantially, the delayed aggregation kinetics and fragmented SEM morphology suggest that inhibitors impair early fibril assembly and structural maturation.

MD simulations further reinforced this mechanism by revealing how PB interacts with aggregation‐prone NAC sequences at the atomic level. Using 1‐μs trajectories and unbiased, coil‐like starting structures, the simulations showed that PB alters hydrogen bonding patterns and interacts with key hydrophobic segments of NAC within the simulation timescale. Residue‐level contact analysis confirmed that PB interacts most robustly with NAC11, spanning A69 to A78, followed by NAC16 and NAC35, consistent with their differential sensitivity to PB in aggregation assays.

These observations are consistent with the greater sensitivity of NAC11 and NAC16 to PB observed in the aggregation assays. In contrast, the extended sequence in NAC35 shows a more distributed interaction pattern, which may contribute to differences in inhibitor response observed experimentally. These structural observations reinforce the functional relevance of the broader A69–A78 region, which overlaps with the 68–71 (GAVV) motif, as a targetable nucleation determinant, and are consistent with the dual effects observed experimentally of PB on nucleation and elongation.

While truncated peptides are powerful tools for dissecting sequence‐specific contributions to aggregation, they simplify the broader aggregation process and do not capture the complexity of full‐length αSyn, where long‐range inter‐domain interactions further modulate aggregation dynamics. Nonetheless, our simulations show that PB alters peptide–peptide interaction patterns and that NAC peptides remain more spatially separated during the simulation period. These findings complement the experimental data by highlighting how PB interacts with specific NAC regions *in silico*. While our extended single‐trajectory simulations yielded consistent and interpretable trends across NAC variants, we acknowledge that the inclusion of replicate runs would strengthen statistical confidence and should be considered in future work. Building on these insights, further work incorporating full‐length αSyn, site‐specific mutagenesis across NAC, and simulations of higher‐order assemblies will be essential to evaluate how early inhibitory interactions affect later stages of fibril propagation.

Collectively, this work advances the understanding of αSyn aggregation by integrating biochemical, structural, and computational approaches and highlights 68–71 (GAVV) and its flanking residues as functionally central and therapeutically targetable elements in synucleinopathies.

## Materials and methods

### NAC peptides and recombinant αSyn

NAC35, NAC17, NAC16, NAC12, NAC11, and NAC8 peptides were custom‐designed and synthesized by GenScript Biotech (Piscataway, NJ, United States) with a purity of ≥ 95%. The lyophilized peptides were dissolved in sterile DMSO according to the manufacturer's instructions and stored at −80 °C until further use.

Inhibitor peptides (PD, PL, and PB) and the scrambled control peptide were custom‐synthesized by Mimotopes (Mulgrave, Victoria, Australia) using standard Fmoc solid‐phase peptide synthesis on automated synthesizers. Peptides were purified by preparative HPLC and verified by electrospray mass spectrometry. Each peptide was supplied with a certificate of analysis, including HPLC and MS profiles with a purity of > 95%. The inhibitor peptides were dissolved in sterile MilliQ water and stored at −20 °C until use. The expression and purification of recombinant human αSyn (A53T) protein were done as described previously [20].

### Hydrophobicity analysis

Hydropathy analysis of NAC peptides was performed using the Kyte‐Doolittle hydropathy scale [21] via the ProtScale tool (ExPASy). Different window sizes were selected based on peptide length to balance the smoothing of hydropathy profiles with resolution: window size 7 for longer peptides (NAC35, NAC17, NAC16), window size 5 for intermediate peptides (NAC12, NAC11), and window size 3 for the shortest peptide (NAC8). Positive hydropathy values indicate hydrophobic residues, while negative values reflect hydrophilic residues.

### Aggregation kinetics

NAC peptides stored at −80 °C were thawed on ice and centrifuged at 12 100 ***g*** for 45 s. A reaction mixture (40 μL) was prepared on ice containing 100 μm NAC peptide, either alone or in combination with inhibitor peptides at a 1 : 2 molar ratio, along with 15 μm ThT (Sigma‐Aldrich, Burlington, MA, USA; Cat. # T3516‐5G) in 1× PBS (pH 7.2). This mixture was then dispensed into a clear‐bottom, black 384‐well plate (Revvity, Waltham, MA, USA), and the plate was sealed with a TopSealA‐PLUS (Revvity) to prevent evaporation.

The aggregation kinetics was monitored on an EnSpire Multimode Plate Reader (Revvity) at 37 °C with a constant agitation of 1000 rpm. To prevent condensation in the assay plate, the upper heater temperature in the plate reader was set to 2 °C higher than the lower heater temperature. ThT (Ex: 460–490 nm, Em: 500–550 nm) fluorescence readings were recorded every 5–10 min for 48–72 h. The aggregation lag time was determined by fitting a Boltzmann sigmoidal equation to the fluorescence data in OriginPro 2024 (OriginLab Corporation, Northampton, MA, USA).

For monitoring the effect of inhibitor peptides on αSyn aggregation, recombinant αSyn was incubated with PD, PL, PB, or scrambled peptides at a 1 : 2 molar ratio (100–150 μm αSyn:200–300 μm inhibitor) in the presence of 50 μm ThT. The incubation was performed at 37 °C with constant agitation at 850 rpm in a Turbo Thermo Shaker (TMS‐200; Allsheng, Hangzhou, China). The formation of amyloid fibrils was monitored by measuring ThT fluorescence over 72 h, as described previously [20].

### Fractionation and quantification of fibril concentration

To fractionate aggregated samples (recombinant αSyn or NAC peptides), 20% of the aggregation mixture was set aside as the ‘total fraction’. The remaining sample was centrifuged at room temperature at 65 000 ***g*** for 1 h. The supernatant was collected as the ‘soluble fraction’ and transferred to a fresh tube. The pellet was resuspended in 80 μL of 1× PBS (pH 7.2) and centrifuged at 65 000 ***g*** for 1 h. After removing the supernatant, the pellet was resuspended in 8 μL of 1× PBS (pH 7.2), yielding a 10× concentrated ‘insoluble fraction’.

The concentration of the total fraction was measured using the Pierce™ BCA Protein Assay Kit (Thermo Fisher Scientific Inc., Waltham, MA, USA, Cat. # 23225). To estimate αSyn fibril concentration in the insoluble fraction, the decrease in monomer concentration in the soluble fraction was quantified by measuring the concentration before and after aggregation. The difference was used to estimate the concentration of fibrils formed.

### ANS assay

A 40‐μL reaction mixture was prepared on ice containing 100 μm NAC peptides and 40 μm 8‐anilino‐1‐naphthalenesulfonic acid (ANS; Sigma‐Aldrich, Cat. # A1028‐5G) in 1× PBS at pH 4.2, 7.2, or 8.0. The aggregation kinetics were monitored on an EnSpire Multimode Plate Reader (Revvity) at 37 °C with a constant agitation of 1000 rpm. ANS fluorescence readings (Ex: 390 nm, Em: 475 nm) were recorded every 5–10 min for 48–72 h. The lag time of aggregation was determined by fitting a Boltzmann sigmoidal equation, as described in the previous section.

### UV–vis spectrophotometric analysis

NAC peptides (100 μm) were incubated without ThT for 48 h to allow aggregation. The resulting mixtures were centrifuged at 65 000 ***g*** for 1 h to separate the soluble and insoluble fractions, as outlined above. Insoluble fractions were resuspended in an equal volume of 1× PBS (pH 7.2) as the soluble fractions. UV–Vis absorption spectra were recorded from 400 to 600 nm using an EnSpire Multimode Plate Reader (Revvity) with a resolution of 10 nm.

### Atomic force microscopy

NAC peptide aggregates, collected after 48 h of the aggregation assay, were dropped onto a freshly cleaved 10 mm AFM mica disc (Ted Pella, Inc., Redding, CA, USA) and air‐dried for 10 min at room temperature. The mica discs were washed five times with deionized water and then dried for 10–15 min at room temperature. AFM imaging was performed as described [36], using the SCANASYST AIR probe (tip radius: 2 nm, spring constant: 0.4 N/m).

### Transmission electron microscopy

Samples were dropped on glow‐discharged carbon‐coated copper grids and negatively stained with 2% uranyl acetate. Electron microscopy was performed on a Tecnai G2 F20 microscope (FEI Technologies, Hillsboro, OR, USA) with an Eagle 4K CCD camera (FEI Technologies) or a CM100 TWIN Transmission Electron Microscope (Philips, Amsterdam, Netherlands) equipped with a side‐mounted Veleta Camera (Olympus, Tokyo, Japan) and processed in the iTEM software suite (Olympus). After bringing the images to 2048 × 2048 pixels, the pixel size at the specimen level was 1.14 nm. Approximately 20 micrographs of each specimen were recorded and evaluated.

### *In vitro* seeding assay

For the *in vitro* seeding assay, αSyn (100 μm), NAC35 (100 μm), NAC16 (200 μm), and NAC11 (200 μm) peptides were aggregated without ThT for 72–96 h. Fibril formation was confirmed by mixing 10 μL of the aggregated sample with 15 μm ThT and measuring fluorescence using an EnSpire Multimode Plate Reader (Revvity). Aggregates were then centrifuged to separate insoluble fractions as described above.

αSyn (100 μm) was incubated with 25 μm insoluble fractions (preformed fibrils) of αSyn, NAC35, NAC16, and NAC11 in a 100–150 μL volume per well of a 96‐well ViewPlate in the presence of 50 μm ThT. The aggregation kinetics were monitored on an EnSpire Multimode Plate Reader (Revvity) at 37 °C with a constant agitation of 1000 rpm. ThT fluorescence was recorded every 5–10 min for over 60–65 h. After aggregation, the contents of each well were collected, and insoluble fractions were isolated by centrifugation. These fractions were subsequently analyzed using dot blot analysis and Coomassie gel staining.

### Coomassie gel staining

The samples were mixed with 2× Laemmli buffer [62.5 mm Tris/HCl (pH 6.8), 25% (w/v) glycerol, 2% SDS, and 0.01% Bromophenol Blue, supplemented with 5% (v/v) 2‐mercaptoethanol] and boiled at 95 °C for 5 min. The samples were then subjected to electrophoresis using Mini‐PROTEAN TGX 4–20% Precast gels (Bio‐Rad, Hercules, CA, USA, Cat. # 4561095) in Tris/glycine/SDS running buffer (Bio‐Rad). Gels were fixed in 50% methanol (v/v) and 10% acetic acid (v/v) for 1 h, stained with Coomassie Brilliant Blue R‐250 Staining Solution (Bio‐Rad, Cat. # 1610436EDU) for 12 h at 4 °C with gentle agitation, and destained in deionized water. Gel images were acquired using a ChemiDoc MP imaging system (Bio‐Rad), and densitometry was performed using the imagej software (RRID: SCR_003070; Bethesda, MD, USA).

### Dot blot analysis

Five μL of the sample (2.5 μL first, allowed to dry completely, then another 2.5 μL) was spotted onto a nitrocellulose membrane (Bio‐Rad, Cat. # 1620147), pre‐divided into uniform circular grids, and allowed to dry for 10 min at room temperature. The membrane was blocked for 1 h with 5% bovine serum albumin (BSA) in 1× Tris‐buffered saline with 0.05% Tween® 20 (TBST), followed by 3× washes with TBST. The membrane was then incubated for 1 h with anti‐α‐Synuclein (D37A6) rabbit monoclonal antibody (Cell Signaling Technology, Inc., Danvers, MA, USA, Cat. # 4179, RRID: AB_1904156), which recognizes an epitope surrounding Glu105 in the C‐terminal region of αSyn, and was used at a 1 : 2000 dilution in 5% BSA/TBST. After 3× washes with TBST, the blot was incubated with Alexa Fluor™ 647 Goat anti‐Rabbit IgG (Thermo Fisher Scientific; Cat. # A‐21245) at a 1 : 5000 dilution for 1 h. The blot was imaged using the ChemiDoc MP imaging system (Bio‐Rad), and densitometry was performed using imagej.

### Microscale thermophoresis

The binding affinity of the inhibitor peptide was measured by microscale thermophoresis (MST) using a Nanotemper Monolith NT.115 instrument (Nanotemper Technologies GmbH, Munich, Germany). Recombinant αSyn was freshly labeled with the Monolith His‐Tag RED tris‐NTA labeling dye according to the manufacturer's protocol (Nanotemper Technologies GmbH). Measurements were conducted in MST buffer (50 mm Tris, 250 mm NaCl, pH 7.0) using standard capillaries (K002; Nanotemper Technologies GmbH). The final concentration of the labeled αSyn in the assay was 50 nm. Binding reactions were incubated on ice for 5 min; then centrifuged at 20 000 ***g*** before loading into the standard glass capillaries (Monolith NTCapillaries, Nano Temper Technologies GmbH). All the measurements were performed with the LED set to 20% intensity and the MST power set to 50%. The laser on‐time was 30 s, and the off‐time was 5 s.

### Scanning electron microscopy of NAC samples

NAC peptides aggregated in the absence or presence of inhibitor peptides, as described in the section Aggregation Kinetics, were collected and pooled into Eppendorf tubes. Approximately 2 μL of each sample was drop‐cast onto a silicon wafer and air‐dried at room temperature. No fixation or sputter coating was applied before imaging.

SEM imaging was performed using a Zeiss Sigma 360 VP microscope (Carl Zeiss Microscopy GmbH, Oberkochen, Germany) at an accelerating voltage of 1 kV with a working distance of approximately 2 mm, under high vacuum conditions. Images were acquired using an InLens secondary electron detector at magnifications of approximately 115× and 958×, selected based on fibril morphology. Aperture settings remained consistent across all samples. Post‐acquisition, brightness and contrast were adjusted uniformly across images to enhance visibility while preserving structural integrity.

### Cell seeding assay

HEK293T αSyn (A53T) CFP/YFP biosensor cells (HEK293T parental line, RRID:CVCL_0063) were kindly provided by Dr. Marc Diamond from the University of Texas Southwestern Medical Center (Dallas, TX, USA). The cells were not further authenticated but were routinely tested and confirmed to be mycoplasma‐free using PCR‐based assays. The cells were maintained in complete growth media consisting of 88% Dulbecco's modified Eagle's growth medium (Lonza, Walkersville, MD, USA, Cat. # 12‐604F), supplemented with 10% (v/v) fetal bovine serum (Thermo Fisher Scientific Inc., Cat. # A5256701), 10 mm HEPES (Serana Europe GmbH, Brandenburg, Germany; Cat # BSL‐001‐100ML), 1% (v/v) GlutaMAX™ Supplement (Thermo Fisher Scientific Inc., Cat. # 35050061), and 1% (v/v) Penicillin–Streptomycin Solution (Thermo Fisher Scientific Inc., Cat. # 15140130). Cells were maintained at 37 °C in a humidified incubator with 5% CO_2_/atmospheric air.

Cells were plated in 96‐well Phenoplate (Revvity, Cat. #6057802; 5000 cells per 100 μL) or 24‐well plate (20 000 cells per 1 mL) in complete growth media. Both plates were pre‐coated overnight with 50 μg·mL^−1^ Poly‐ᴅ‐Lysine (Sigma‐Aldrich, Cat. # P6407). The following day, cells were transfected with the total fraction (containing both monomers and fibrils) or the insoluble fraction (containing primarily fibrils) of the αSyn aggregation reaction. These fractions were prepared by aggregating αSyn with or without inhibitors. Transfection mixtures were prepared by combining 1 μm of the total fraction or insoluble fraction, or an equivalent concentration of only inhibitor peptides, in 20 μL (for 96‐well plate) or 100 μL (for 24‐well plate) of Opti‐MEM™ I Reduced Serum Medium (Thermo Fisher Scientific Inc., Cat. #11058021), supplemented with 0.5 μL (96‐well plate) or 4 μL (24‐well plate) TurboFect™ Transfection Reagent (Thermo Fisher Scientific Inc., Cat. # R0531). Controls included mixtures with Opti‐MEM and TurboFect™ but without αSyn or Opti‐MEM and αSyn but without TurboFect™. Before preparation of the transfection mixtures, αSyn samples were sonicated using Branson Ultrasonic™ Sonifier Cup Horns (Marshall Scientific, Hampton, NH, USA) with settings: 50% amplitude, 30 s ON, 10 s OFF, for a total of 1 min, to break fibrils into smaller fragments. The transfection mixture was briefly vortexed and incubated at room temperature for 15 min before adding to the cells. Twenty milliliters (for 96‐well plate) or 100 μL (for 24‐well plate) of transfection mixture was added dropwise to the cells, and the plate was gently tapped to mix the contents. After 72 h, cells were either fixed directly in the plates for imaging or harvested using 0.5% Trypsin–EDTA (Thermo Fisher Scientific Inc., Cat. # 15400054) and then fixed for flow cytometric analysis. Fixed cells in 96‐well plates were washed once with 1× PBS and stained with 10 μm Hoechst‐33 342 nuclear dye (Invitrogen, Carlsbad, CA, USA, Cat. #H21492) diluted in PBS for 10 min at room temperature. To preserve the cells for imaging, a 1% glycerol solution prepared in deionized water was added to each well. For flow cytometric analysis, trypsinized cells from 3 wells per experiment condition were pooled, fixed with 2% paraformaldehyde, washed once with PBS, and then resuspended in PBS for further processing.

### Quantification of CFP/YFP inclusions

Cells were imaged using a high‐content imaging system (Cell Voyager 7000S; Yokogawa, Tokyo, Japan) with a 20× objective. CFP/YFP inclusions of αSyn (A53T) were detected using a 488 nm laser line (Ex = 460–490 nm, Em = 500–550 nm), while Hoechst‐33 342‐stained nuclei were detected using the 405 nm laser line (Ex = 360–400 nm, Em = 410–480 nm). At least 10 focal areas per well were imaged for each experimental replicate. Acquired images were processed using Signals Image Artist (Revvity). The number of cells quantified by Hoechst‐stained nuclei and the number of CFP/YFP inclusions used to quantify intracellular seeding were analyzed using an image analysis script in Signals Image Artist (see Appendix S1). The total number of inclusions per well was normalized to cell confluency.

### Flow cytometry analysis cell seeding

Cells were analyzed on a FACSAria II Sorpe flow cytometer (BD Biosciences, Franklin Lakes, NJ, USA). Initially, 50 000 cells were acquired to set up gating. Dead cells and debris were excluded using forward scatter versus side scatter gating. Intact cells were then gated and analyzed in the blue/green channels. The gating strategy to identify cells with aggregates (P2 gate) was based on the expression levels of fluorescent protein tags (CFP/YFP). YFP was excited with a 488 nm laser, and emission was detected by a 525/50 nm (green) bandpass filter. CFP was excited with a 445 nm laser, and emission was detected by a 530/30 nm (blue) bandpass filter. The integrated FRET density was calculated by multiplying the percentage of FRET‐positive cells by their median fluorescence intensity within the FRET‐positive gate, as previously described [24]. This density was then normalized for treated groups relative to untreated controls (cells not exposed to fibrils). Data analysis was performed using BD FACSDiva™ Software.

### MD simulation

MD simulations were conducted using the GROMACS package with parameters from the GROMOS‐53A6 force field [37, 38]. Initial peptide structures were obtained from the AlphaFold3 web server [39] and subjected to a 100 ns equilibration simulation to allow relaxation into coil‐rich conformations, appropriate for disordered NAC peptides in aqueous solution. Final conformations from this pre‐simulation step were used to initiate the main 1000 ns production runs. Each system consisted of equimolar amounts of PB and a given NAC peptide (NAC35, NAC16, or NAC11), with 10 PB and 10 NAC peptides randomly placed in a cubic simulation box (~ 10 × 10 × 10 nm^3^), ensuring a minimum 1.0 nm distance between peptides and box edges to avoid interactions from periodic images. Systems were solvated using the SPC/E water model [40], electrically neutralized, and energy minimized using the steepest descent algorithm until the maximum force on atoms was < 10 kJ·mol^−1^·nm^−1^. Temperature was maintained at 300 K using the V‐rescaling thermostat [41] and pressure at 1 bar using the Parrinello‐Rahman barostat [42]. Short‐range non‐bonded interactions were calculated with a 1.2 nm cutoff, and long‐range electrostatics were handled using the Particle Mesh Ewald method. A 2‐fs time‐step was used for all simulations. Simulation trajectories were analyzed using GROMACS utilities and vmd software [43]. A detailed summary of each system's composition and simulation setup is provided in Table S1.

### Statistics analysis

Data are presented as mean ± SEM (*n* ≥ 2 independent experiments, unless stated otherwise). One‐way ANOVA tests were utilized to compare means across multiple groups within a single factor. Multiple comparisons for ANOVA tests were performed using Tukey's. Statistical significance was set at *P* < 0.05. *F* values and degrees of freedom for all ANOVA analyses are summarized in Tables S2–S6.

## Conflict of interest

The authors declare no conflict of interest.

## Author contributions

VD: conceptualization, methodology, investigation, interpretation, funding acquisition, supervision, and writing – original draft, review, and editing. SMMM: methodology, investigation. NA: methodology, investigation. SS: methodology and investigation. FM: methodology and investigation. SM: methodology and investigation. LM: methodology and investigation. MK: methodology and investigation. VR: investigation, interpretation, visualization. IF: methodology and investigation. RK: methodology and investigation. SH: methodology, investigation, and interpretation. MH: resources and funding acquisition. MN: methodology, investigation, interpretation, funding acquisition, and writing – original draft, review, and editing.

## Supporting information


**Fig. S1.** ThT fluorescence and AFM images of NAC aggregates.
**Fig. S2.** UV‐Vis analysis of NAC peptide total fractions.
**Fig. S3.** Raman spectroscopy confirms aggregation‐induced β‐sheet formation in NAC35, NAC16, and NAC11 fibrils.
**Fig. S4.** ThT kinetics of preformed fibrils.
**Fig. S5.** Dot blot image and Coomassie‐stained gel image for Fig. 3.
**Fig. S6.** MST traces and dose‐response curves for αSyn binding to inhibitor peptides.
**Fig. S7.** TEM images of αSyn fibrils formed with scrambled peptides.
**Fig. S8.** ThT kinetics of inhibitor peptides alone.
**Fig. S9.** Original uncropped image of the dot blot shown in Fig. 4f.
**Fig. S10.** Coomassie‐stained gels for Fig. 4h.
**Fig. S11.** CFP/YFP inclusion quantifications and FRET flow cytometry side scatter plot.
**Fig. S12.** High‐magnification SEM images of NAC11 fibrils.
**Fig. S13.** Scanning electron microscope (SEM) images of inhibitor peptides PD, PL, and PB.
**Table S1.** Details of the components of the MD simulation systems.
**Table S2.** F values and degrees of freedom for one‐way ANOVA performed in Fig. 1c, h‐j.
**Table S3.** F values and degrees of freedom of one‐way ANOVA performed in Fig. 3b and d.
**Table S4.** F values and degrees of freedom of one‐way ANOVA performed in Fig. 4b, d, k, and l.
**Table S5.** F values and degrees of freedom of one‐way ANOVA performed in Fig. 5c‐e.
**Table S6.** F values, degrees of freedom, and other details of one‐way ANOVA performed in Fig. 6D‐G.
**Appendix S1.** Image Analysis Script to quantify CFP/YFP inclusions and cell confluence.

## Data Availability

Source data supporting this study are available in Zenodo at DOI: 10.5281/zenodo.15271406, including raw experimental and simulation data for Figs 1, 2, 3, 4, 5, 6, 8, 9, 10, and Figs S2–S4. Additional data supporting the findings are provided within the article and its Supplementary Materials.
