# Supplementary material for: Hydrophobic residues in the α‐synuclein NAC domain drive seed‐competent fibril formation and are targeted by peptide inhibitors
Source: FEBS J. 2025 Aug 17;293(1):134–55. doi: 10.1111/febs.70222 (PMC12797002; doi:10.1111/febs.70222)
Supplement: Supplementary file 1 — Fig. S1. ThT fluorescence and AFM images of NAC aggregates. Fig. S2. UV‐Vis analysis of NAC peptide total fractions. Fig. S3. Raman spectroscopy confirms aggregation‐induced β‐sheet formation in NAC35, NAC16, and NAC11 fibrils. Fig. S4. ThT kinetics of preformed fibrils. Fig. S5. Dot blot image and Coomassie‐stained gel image for Fig. 3. Fig. S6. MST traces and dose‐response curves for αSyn binding to inhibitor peptides. Fig. S7. TEM images of αSyn fibrils formed with scrambled peptides. Fig. S8. ThT kinetics of inhibitor peptides alone. Fig. S9. Original uncropped image of the dot blot shown in Fig. 4f. Fig. S10. Coomassie‐stained gels for Fig. 4h. Fig. S11. CFP/YFP inclusion quantifications and FRET flow cytometry side scatter plot. Fig. S12. High‐magnification SEM images of NAC11 fibrils. Fig. S13. Scanning electron microscope (SEM) images of inhibitor peptides PD, PL, and PB. Table S1. Details of the components of the MD simulation systems. Table S2. F values and degrees of freedom for one‐way ANOVA performed in Fig. 1c, h‐j. Table S3. F values and degrees of freedom of one‐way ANOVA performed in Fig. 3b and d. Table S4. F values and degrees of freedom of one‐way ANOVA performed in Fig. 4b, d, k, and l. Table S5. F values and degrees of freedom of one‐way ANOVA performed in Fig. 5c‐e. Table S6. F values, degrees of freedom, and other details of one‐way ANOVA performed in Fig. 6D‐G. Appendix S1. Image Analysis Script to quantify CFP/YFP inclusions and cell confluence. [file FEBS-293-134-s001.pdf]

Supplementary material

Extending the Role of NAC Domain Hydrophobic Residues in αSynuclein: Implications for Seed-Competent Fibril Formation

Viswanath Das,[1,2,*] Sayed Mostafa Modarres Mousavi,[3] Narendran Annadurai,[1] Sunčica Sukur,[1] Faramarz Mehrnejad,[4] Sajad Moradi,[5] Lukáš Malina,[1,6] Markéta Kolaříková,[6] Vaclav Ranc,[1] Ivo Frydrych,[1] Roman Kouřil,[7] Saman Hosseinkhani,[8] Marián Hajdúch,[1,2] and Maryam Nikkhah[3,*]

[1] Institute of Molecular and Translational Medicine, Faculty of Medicine and Dentistry, Palacký University and University Hospital Olomouc, Hněvotínská 1333/5, 779 00 Olomouc (Czech Republic)

[2] Institute of Molecular and Translational Medicine, Czech Advanced Technologies and Research Institute, Palacký University Olomouc, Křížkovského 511/8, 779 00, Olomouc (Czech Republic)

[3] Department of Nanobiotechnology, Faculty of Biological Sciences, Tarbiat Modares University, Tehran, 14115-154 (Iran)

[4] Department of Nanobiotechnology and Biomimetics, School of Life Science Engineering, University of Tehran, Tehran (Iran).

[5] Nano Drug Delivery Research Centre, Health Technology Institute, Kermanshah University of Medical Sciences, Kermanshah (Iran)

[6] Department of Medical Biophysics, Faculty of Medicine and Dentistry, Palacký University Olomouc, Hněvotínská 3, 775 15 Olomouc (Czech Republic)

[7] Department of Biophysics, Faculty of Science, Palacký University Olomouc, Šlechtitelů 27, 783 71 Olomouc (Czech Republic)

[8] Department of Biochemistry, Faculty of Biological Sciences, Tarbiat Modares University, Tehran, 14115-154 (Iran)

***Correspondence**: viswanath.das@upol.cz (V.D.), m_nikkhah@modares.ac.ir (M.N.).

SUPPLEMENTARY FIGURES, RESULTS AND METHOD

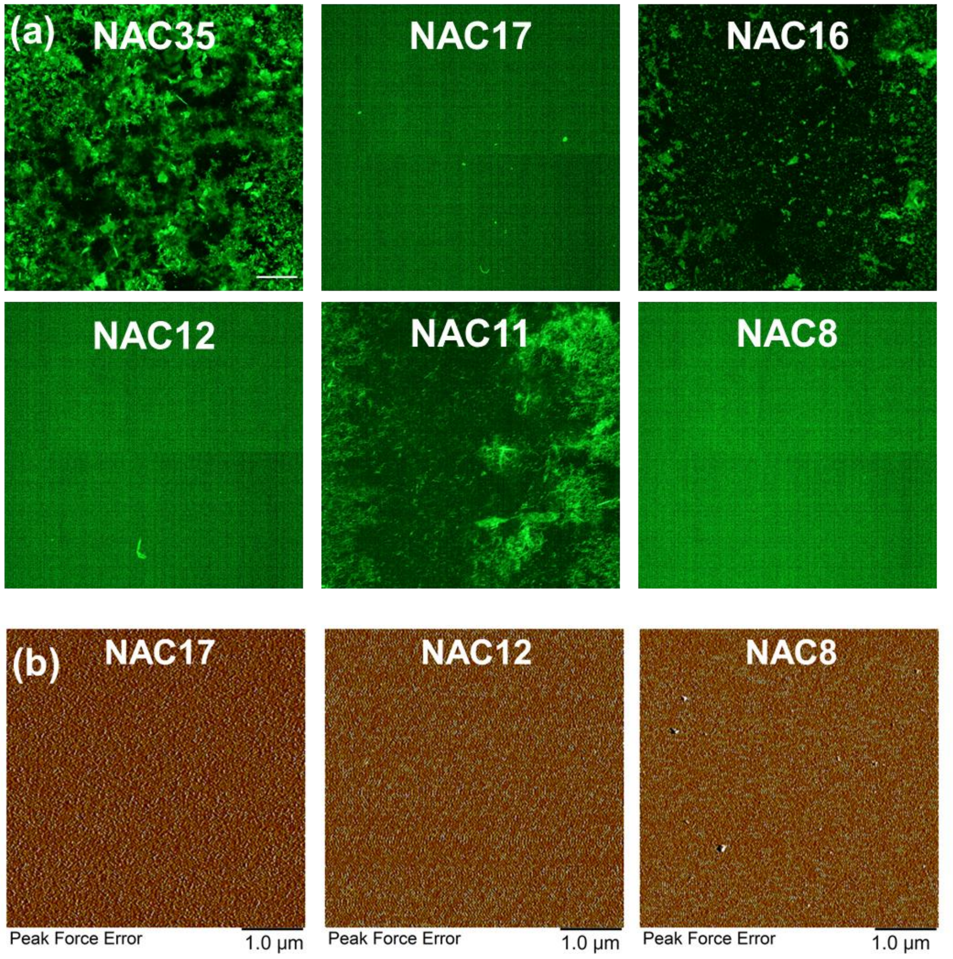

Figure S1. ThT fluorescence and AFM images of NAC aggregates.

NAC peptides (100 µM) were aggregated for 48 h with ThT. After the assay, wells were embedded in 0.05% low-melting agarose, solidified at room temperature, and imaged with a 4x objective on a Cell Voyager CV7000S microscope (Yokogawa) using a 488 nm laser (Ex = 460-490 nm, Em = 500-550 nm). (a) Fluorescence images of ThT-stained NAC aggregates after 48 h of aggregation. Scale bar: 200 µm. (b) AFM images of NAC17, NAC12, and NAC8 samples collected from assay wells after 48 h of aggregation show the absence of aggregates. Scale bar: 1 µm

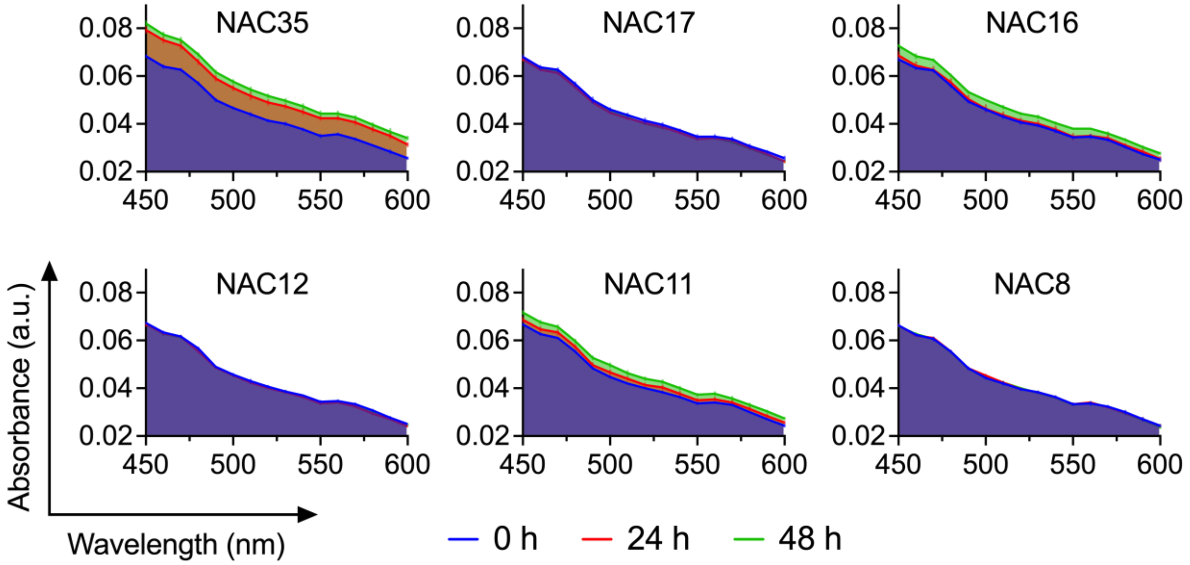

Figure S2. UV-Vis analysis of NAC peptide total fractions.

NAC peptides (100 µM) were aggregated without ThT for 48 h. Samples were collected at 24 h intervals and transferred to a 384-well SpectraPlate (Revvity) for absorbance measurements as described in the Methods section. UV-Vis absorption spectra of NAC peptides in their monomeric form (0 h) and after 24-48 h of aggregation. The 0 h sample reflects unaggregated monomers. Data represent the means of 3 replicates. NAC peptides (100 µM) were aggregated without ThT for 48 h. Samples were collected at 24 h intervals and transferred to a 384-well SpectraPlate (Revvity) for absorbance measurements as described in the Methods section. Source data is available in Zenodo at DOI: 10.5281/zenodo.15271406.

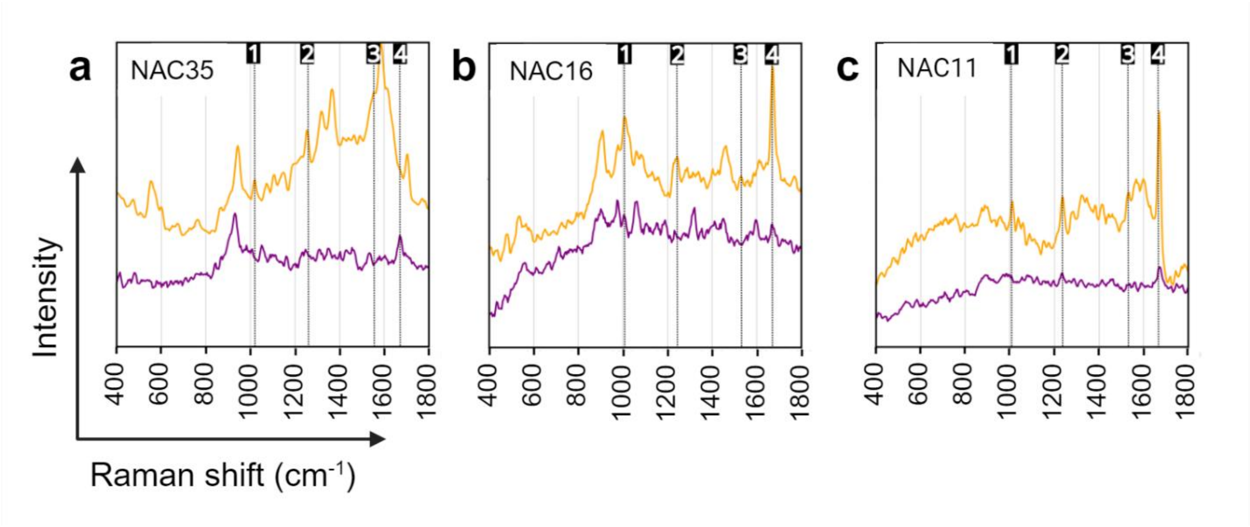

Figure S3. Raman spectroscopy confirms aggregation-induced β-sheet formation in NAC35, NAC16, and NAC11 fibrils.

NAC peptide monomers and the samples aggregated for 48 h were subjected to Raman analysis. The Raman system was equipped with a green laser operating at 532 nm with a power of 1 mW measured on the sample. The exposition time was set to 3s, and spectra were averaged from 28 micro-scans. Each sample was placed on CaF_2 microscopy glass (Crystan Ltd., Poole, Dorset, UK) and measured at ten randomly selected spots. The spectral data were processed using a Python script (version 3.11). First, the fluorescent background was removed by subtracting a polynomial function (n = 5), and then the spectra were smoothed using the Savitzky-Golay method.

Raman spectra show structural differences between monomeric (purple trace) and aggregated (orange trace) forms of (a) NAC35, (b) NAC16, and (c) NAC11. In all aggregates, increased amide I (1675 cm^{-1}, labeled 4), amide II (1552 cm^{-1}, labeled 3), and amide III (1252 cm^{-1}, labeled 2) band intensities suggest β-sheet formation. NAC35 exhibits the most pronounced red shift in the amide I band, indicating extensive β-sheet structure, while NAC16 and NAC11 show smaller band broadening, reflecting lower β-sheet content. Additional spectral changes, including skeletal signals (900–1000 cm^{-1}, labeled 1), further confirm aggregation-induced structural rearrangements. Source data is available in Zenodo at DOI: 10.5281/zenodo.15271406.

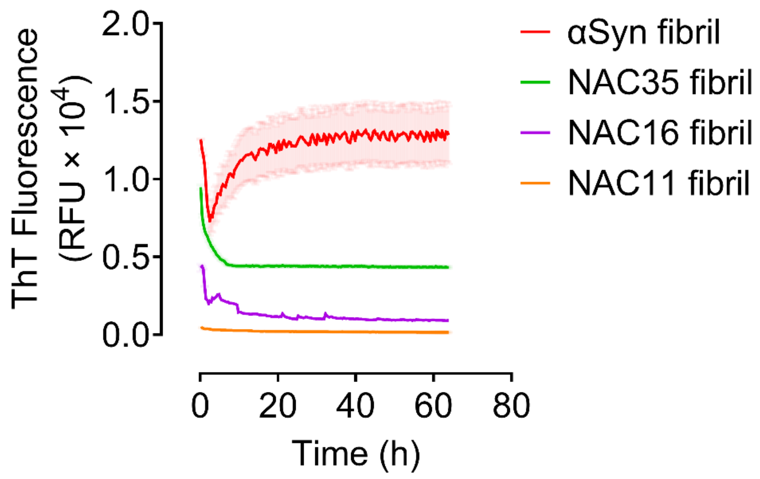

Figure S4. **ThT kinetics of preformed fibrils.**

ThT fluorescence kinetics of preformed fibrils of αSyn, NAC35, NAC16, and NAC11 were monitored using ThT fluorescence under conditions similar to those described for the *In Vitro Seed Assay* in the primary Method [Mean ± SEM (n = 2); Source data is available in Zenodo at DOI: 10.5281/zenodo.15271406.

Briefly, 25 µM insoluble fractions (preformed fibrils) were incubated with 50 µM ThT, but without monomeric αSyn. The aggregation was monitored using an EnSpire Multimode Plate Reader (Revvity) at 37°C with constant agitation at 1000 rpm for over 60-65 h.

Preformed αSyn fibrils displayed an initial stabilization phase, followed by a stable ThT fluorescence signal, indicative of the stationary phase of fibril aggregation. NAC fibrils exhibited high initial ThT fluorescence, likely due to nonspecific ThT binding, which decreased as the system equilibrated and stabilized without further growth.

These kinetic profiles were used to establish baseline correction values for seeded aggregation assays shown in Fig. 3. Baseline correction values were derived from the stable ThT fluorescence observed during the stationary phase of preformed fibril kinetics (approximately 15-20 hours for αSyn and 2-5 hours for NAC fibrils). This correction ensured accurate quantification of seeded aggregation by accounting for the intrinsic fluorescence of preformed fibrils. Unlike αSyn fibrils, which retained the ability to grow through secondary aggregation, NAC fibrils showed no elongation during the observation period.

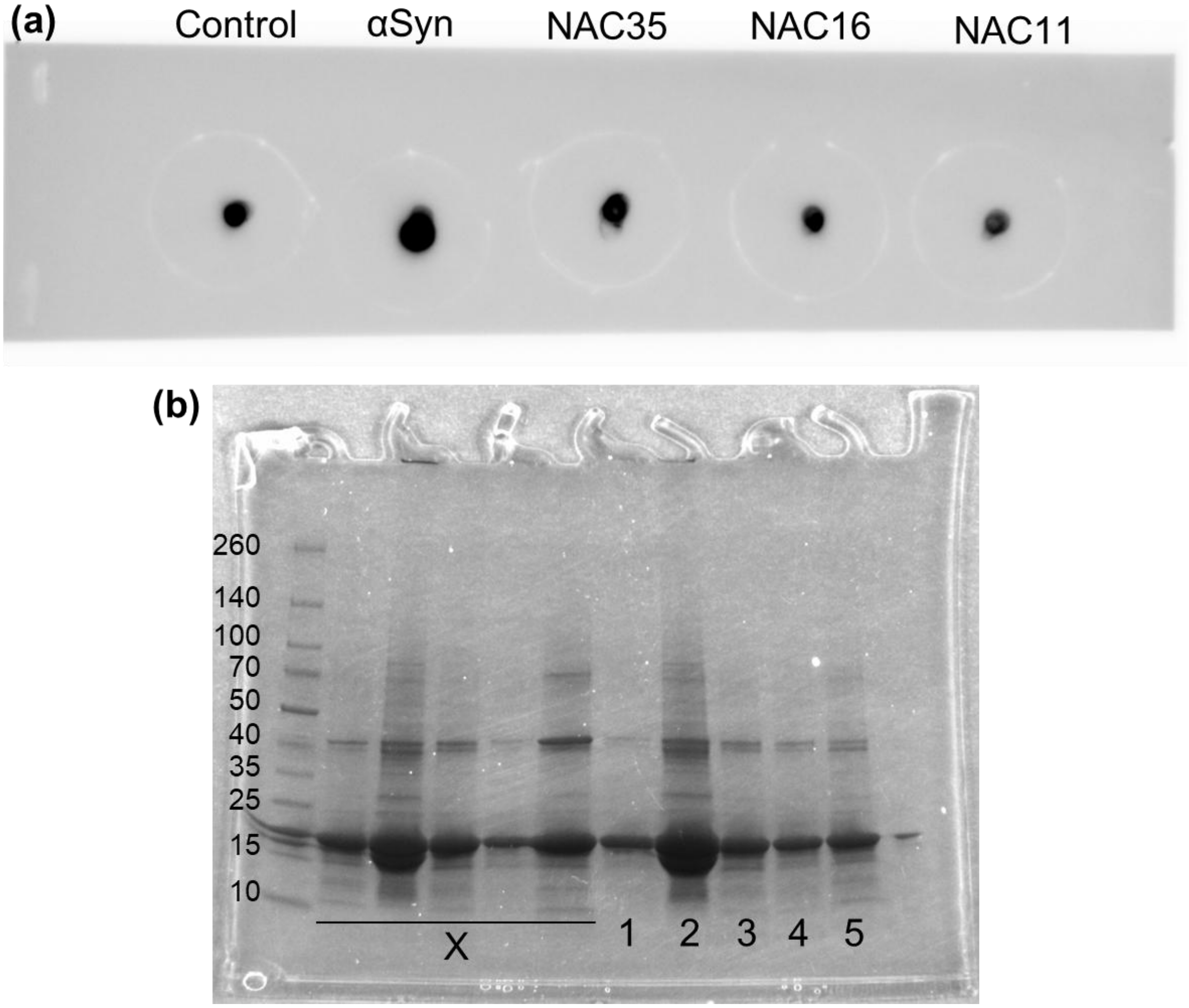

Figure S5. Dot bot image and Coomassie-stained gel image for Figure 3.

(a) Original uncropped image of the dot blot shown in Figure 3b. (b) Image of full-length uncropped gel of insoluble fractions of αSyn aggregated with or without preformed fibrils shown in Figure 3c. Lanes: 1: αSyn, 2: αSyn + αSyn fibril, 3: αSyn + NAC35 fibril, 4: αSyn + NAC16 fibril, and 5: αSyn + NAC11 fibril.

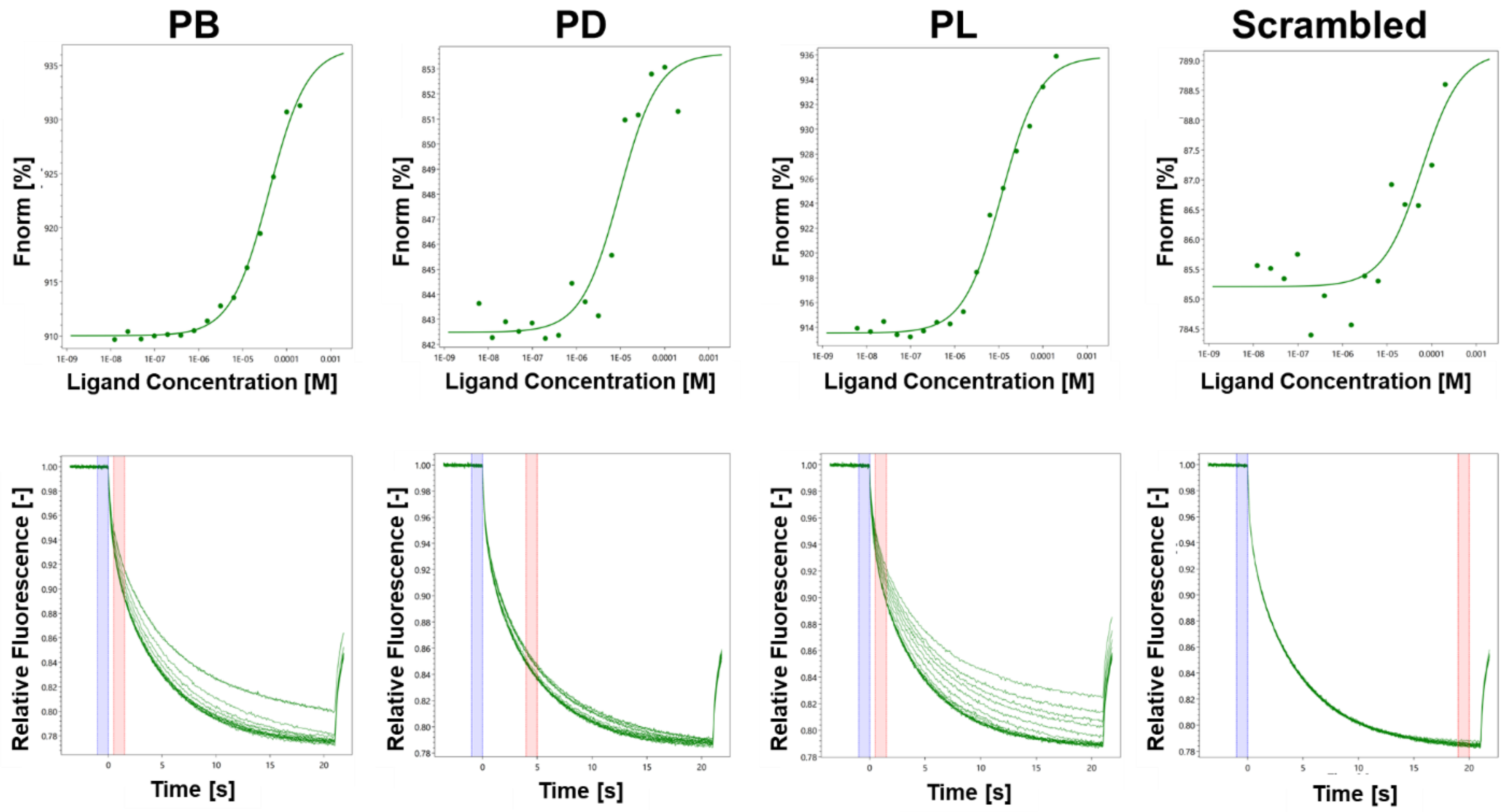

Figure S6. MST traces and dose-response curves for αSyn binding to inhibitor peptides.

MST traces (top) and dose-response curves (bottom) show the binding of His_6-tagged αSyn labeled with RED-tris-NTA to PD, PB, PL, and scrambled peptides. Dose-response curves were fitted to a one-site binding model to determine K_d values. All measurements were performed in triplicate.

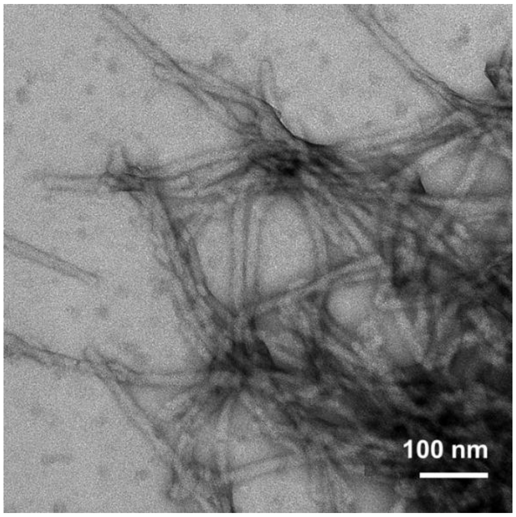

Figure S7. TEM images of αSyn fibrils formed with scrambled peptides.

TEM images of αSyn fibrils formed in the presence of scrambled peptides show no effect on fibril morphology.

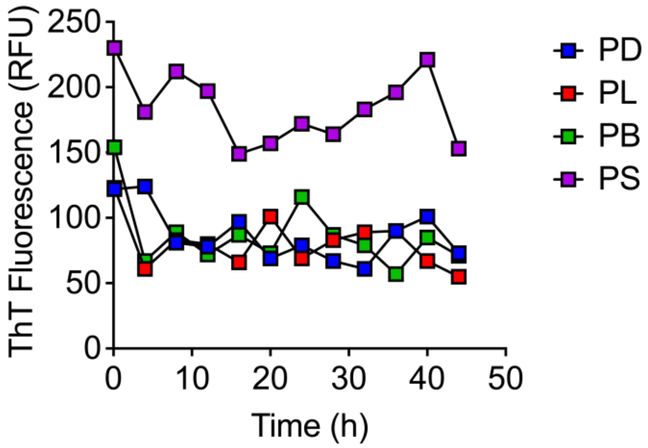

Figure S8. ThT kinetics of inhibitor peptides alone

ThT kinetics data for PD, PL, PB, and PS peptides at 200 μM concentration with 50 μM ThT showed no signs of self-aggregation. N = 1.

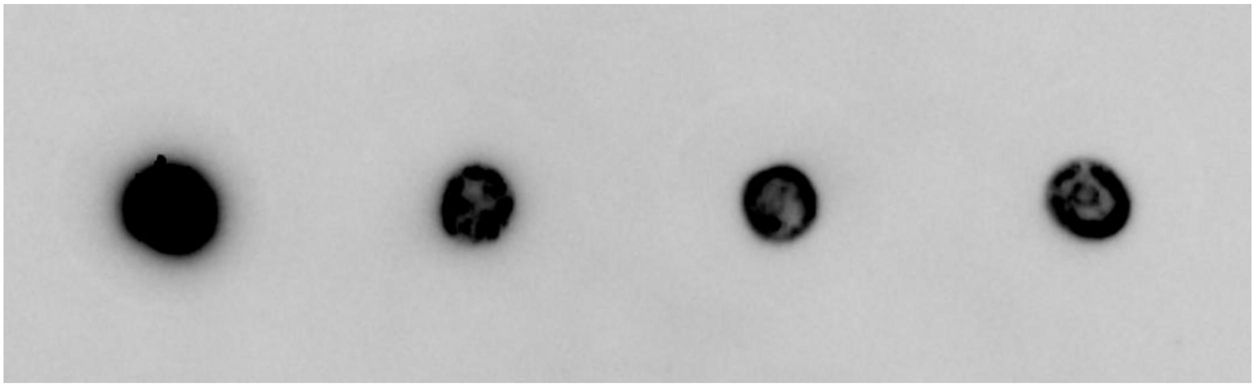

Figure S9. Original uncropped image of the dot blot shown in Figure 4f.

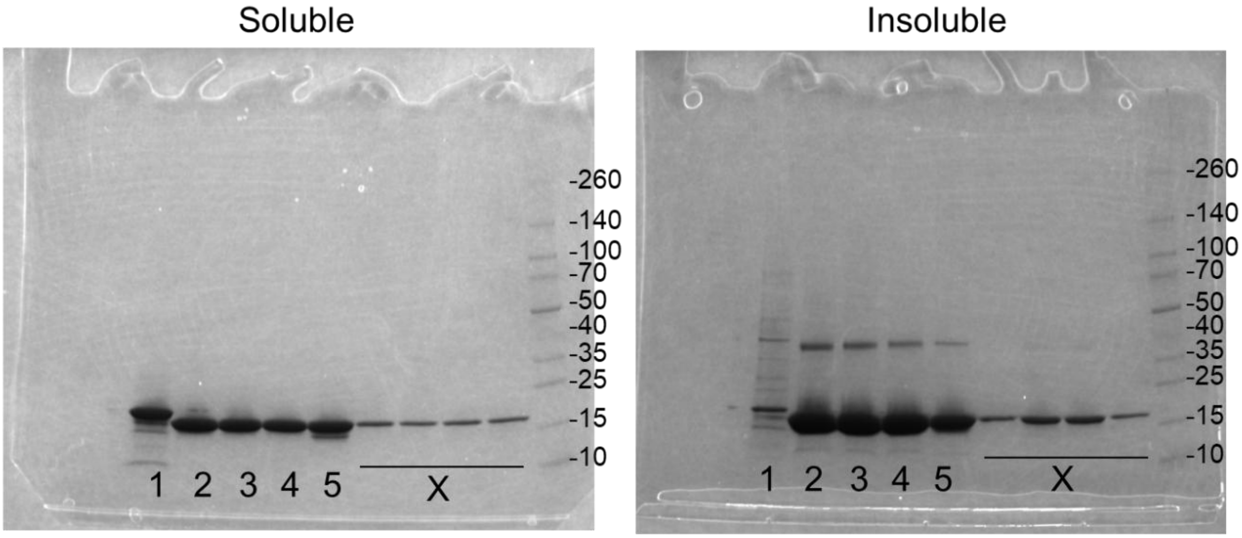

Figure S10. **Coomassie-stained gels for Figure 4h.**

Uncropped Coomassie-stained gels of soluble and insoluble fractions of αSyn aggregated with or without inhibitors. Lanes **1**: Monomer, **2**: αSyn, **3**: αSyn + PD, **4**: αSyn + PL, and **5**: αSyn + PB.

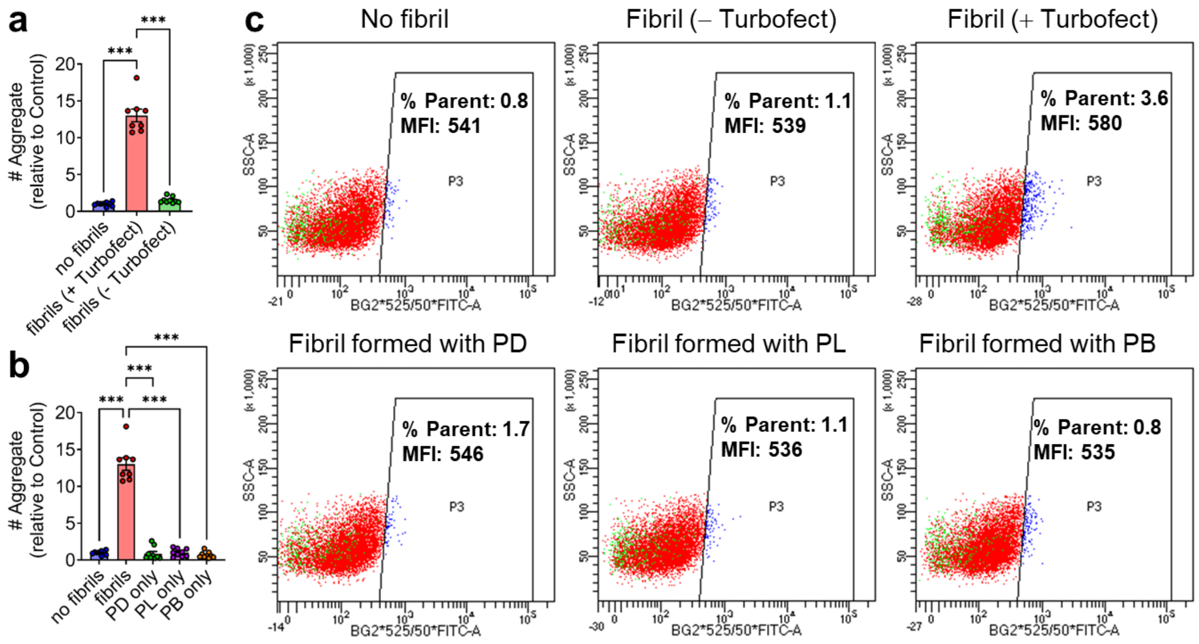

Figure S11. CFP/YFP inclusion quantifications and FRET flow cytometry side scatter plot.

Quantification of CFP/YFP inclusions in biosensor cells transduced with αSyn fibrils from the total fraction with (+) or without (-) Turbofect transfection reagents (**a**) or αSyn fibrils and only PD, PL, and PB inhibitor peptides (**b**). Mean ± SEM (n = 3 independent experiments). ***P ≤ 0.001, One-way ANOVA with Tukey's posthoc test. (**c**) Cells were treated with fibrils formed under various conditions (No fibril, Fibril - TurboFect, Fibril + TurboFect, Fibril formed with PD, PL, or PB). Cells were analyzed for side scatter (SSC-A) and fluorescence in both the CFP (excited at 445 nm) and YFP (excited at 488 nm) channels to detect intracellular seeding and the formation of aggregates. The P3 gate represents cells positive for both CFP and YFP fluorescence, indicating the presence of inclusions. The percentage of the parent population (% Parent) and the median fluorescence intensity (MFI) within the P3 gate are displayed. Fibril transduction with TurboFect resulted in the highest % Parent (3.6%) and MFI (580), suggesting increased intracellular seeding and aggregate formation compared to other conditions.

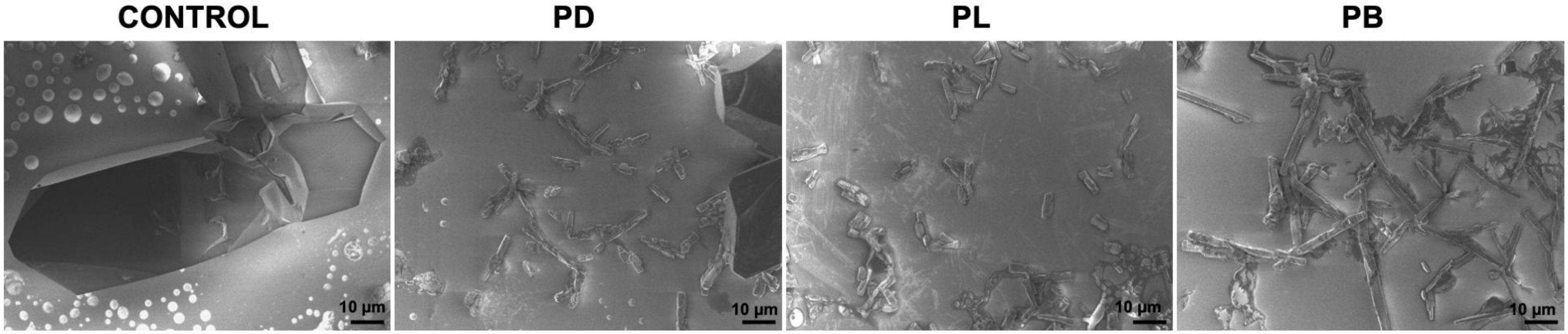

Figure S12. **High-magnification SEM images of NAC11 fibrils.**

SEM images of NAC11 fibrils formed under control conditions and after treatment with PD, PL, or PB, acquired at higher magnification to allow direct comparison with NAC35 and NAC16 shown in Fig. 7. Scale bar: 10 µm.

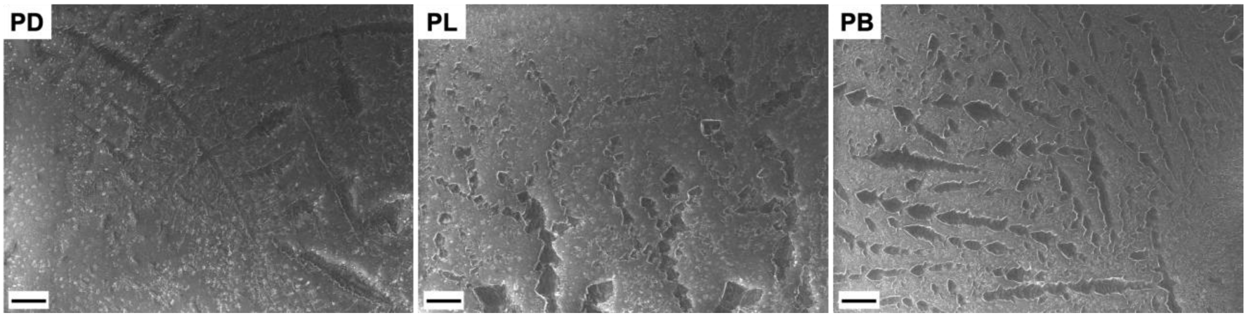

Figure S13. **Scanning electron microscope (SEM) images of inhibitor peptides PD, PL, and PB.**

The peptides were incubated under the same aggregation conditions used for NAC peptides in Figure 6. SEM analysis shows that the inhibitor peptides did not form fibrillar or amorphous aggregates. Scale bar: 10 µm.

SUPPLEMENTARY TABLES

Table S1. Details of the components of the MD simulation systems.

System	Number of Peptides	Box size (nm^3)	Temperature(K^0)	Duration (ns)
**PB**	10	207	300	1000
**NAC11**	10	122	300	1000
**NAC11/PB**	10/10	718	300	1000
**NAC16**	10	214	300	1000
**NAC16/PB**	10/10	980	300	1000
**NAC35**	10	508	300	1000
**NAC35/PB**	10/10	1704	300	1000

Table S2. F values and degrees of freedom for one-way ANOVA performed in Fig. 1c, h-j.

**Fig. 1c**						
	**SS**	**DF**	**MS**	**F (DFn**	**DFd)**	**P value**
Treatment (between columns)	28	2	14	F (2	6) = 149	P<0.001
Residual (within columns)	0.55	6	0.092			
Total	28	8				
Number of treatments (columns)	3					
Number of values (total)	9					
**Fig. 1h**						
	**SS**	**DF**	**MS**	**F (DFn**	**DFd)**	**P value**
Treatment (between columns)	44	2	22	F (2	6) = 251	P<0.001
Residual (within columns)	0.52	6	0.087			
Total	44	8				
Number of treatments (columns)	3					
Number of values (total)	9					
**Fig. 1i**						
	**SS**	**DF**	**MS**	**F (DFn**	**DFd)**	**P value**
Treatment (between columns)	0.12	2	0.062	F (2	6) = 15	P=0.004
Residual (within columns)	0.024	6	0.0041			
Total	0.15	8				
Number of treatments (columns)	3					
Number of values (total)	9					
**Fig. 1j**						
	**SS**	**DF**	**MS**	**F (DFn**	**DFd)**	**P value**
Treatment (between columns)	0.25	2	0.13	F (2	6) = 12	P=0.008
Residual (within columns)	0.062	6	0.01			
Total	0.31	8				
Number of treatments (columns)	3					
Number of values (total)	9					

Table S3. F values and degrees of freedom of one-way ANOVA performed in Fig. 3b and d.

**Fig. 3a**					
	SS	DF	MS	F (DFn, DFd)	P value
Treatment (between columns)	1028	3	342.7	F (3, 8) = 19.62	P<0.001
Residual (within columns)	139.8	8	17.47		
Total	1168	11			
Data summary					
Number of treatments (columns)	4				
Number of values (total)	12				
**Fig. 3d**					
	SS	DF	MS	F (DFn, DFd)	P value
Treatment (between columns)	2.14	4	0.54	F (4, 25) = 7.52	P<0.001
Residual (within columns)	1.78	25	0.07		
Total	3.92	29			
Data summary					
Number of treatments (columns)	5				
Number of values (total)	30				
**Fig. 3e**					
	SS	DF	MS	F (DFn, DFd)	P value
Treatment (between columns)	2.68	4	0.67	F (4, 15) = 14.08	P<0.001
Residual (within columns)	0.71	15	0.05		
Total	3.39	19			
Data summary					
Number of treatments (columns)	5				
Number of values (total)	20				

Table S4. F values and degrees of freedom of one-way ANOVA performed in Fig. 4b, d, k, and l.

**Fig. 4b**						
	SS	DF	MS	F (DFn	DFd)	P value
Treatment (between columns)	3500	3	1167	F (1.0	1.0) = 5972	P=0.008
Individual (between rows)	13	1	13	F (1	3) = 64	P=0.004
Residual (random)	0.59	3	0.2			
Total	3514	7				
Number of treatments (columns)	4					
Number of subjects (rows)	2					
**Fig. 4d**						
	SS	DF	MS	F (DFn	DFd)	P value
Treatment (between columns)	3.5	4	0.87	F (4	9) = 135	P<0.001
Residual (within columns)	0.058	9	0.0065			
Total	3.5	13				
Number of treatments (columns)	5					
Number of values (total)	14					
**Fig. 4g**						
	SS	DF	MS	F (DFn	DFd)	P value
Treatment (between columns)	0.557	3	0.1857	F (3	8) = 14.17	P=0.001
Residual (within columns)	0.1049	8	0.01311			
Total	0.6619	11				
Number of treatments (columns)	4					
Number of values (total)	12					
**Fig. 4**						
	SS	DF	MS	F (DFn	DFd)	P value
Treatment (between columns)	0.6381	3	0.2127	F (3	12) = 9.325	P=0.002
Residual (within columns)	0.2737	12	0.02281			
Total	0.9118	15				
Number of treatments (columns)	4					
Number of values (total)	16					

Table S5. F values and degrees of freedom of one-way ANOVA performed in Fig. 5c-e.

**Fig. 5c**					
	SS	DF	MS	F (DFn, DFd)	P value
Treatment (between columns)	630,1	4	157,5	F (4, 25) = 87.85	P<0.001
Residual (within columns)	44,82	25	1,793		
Total	674,9	29			
Number of treatments (columns)	5				
Number of values (total)	30				
**Fig. 5d**					
	SS	DF	MS	F (DFn, DFd)	P value
Treatment (between columns)	1,253	3	0,4176	F (3, 20) = 50.38	P<0.001
Residual (within columns)	0,1658	20	0,008289		
Total	1,419	23			
Number of treatments (columns)	4				
Number of values (total)	24				

**Fig. 5e**					
	SS	DF	MS	F (DFn, DFd)	P value
Treatment (between columns)	5,401	4	1,35	F (4, 10) = 15.26	P<0.001
Residual (within columns)	0,8849	10	0,08849		
Total	6,286	14			
Number of treatments (columns)	5				
Number of values (total)	15				

Table S6. F values, degrees of freedom, and other details of one-way ANOVA performed in Fig. 6d-g.

**Fig. 6d**					
	SS	DF	MS	F (DFn, DFd)	P value
Treatment (between columns)	86,22	3	28,74	F (3, 12) = 569.9	P<0.001
Residual (within columns)	0,6052	12	0,05043		
Total	86,82	15			
Number of treatments (columns)	4				
Number of values (total)	16				
**Fig. 6e**					
	SS	DF	MS	F (DFn, DFd)	P value
Treatment (between columns)	95,57	3	31,86	F (3, 12) = 81.14	P<0.001
Residual (within columns)	4,711	12	0,3926		
Total	100,3	15			
Number of treatments (columns)	4				
Number of values (total)	16				

**Fig. 6f**				
	SS	DF	MS	F (DFn, DFd)
Treatment (between columns)	34,76	3	11,59	F (3, 16) = 99.81
Residual (within columns)	1,857	16	0,1161	
Total	36,61	19		
Number of treatments (columns)	4			
Number of values (total)	20			
**Fig. 6g**				
	SS	DF	MS	F (DFn, DFd)
Treatment (between columns)	0,913	3	0,3043	F (3, 12) = 20.12
Residual (within columns)	0,1815	12	0,01513	
Total	1,094	15		
Number of treatments (columns)	4			
Number of values (total)	16			

APPENDIX

Appendix S1. **Image Analysis Script to quantify CFP/YFP inclusions and cell confluence**. This appendix illustrates the step-by-step procedure for applying the Image Analysis Script to quantify intracellular CFP/YFP inclusions and cell confluence. The steps are demonstrated using example images of control cells (fibril untransduced) and cells transduced with 1 μM αSyn fibrils after 72 hours of the seeding experiment. In control (untransduced) cells, only basal levels of CFP/YFP inclusions are detected, reflecting the natural background signal. In contrast, cells transduced with αSyn fibrils exhibit higher CFP/YFP inclusion levels, confirming successful seeding. Each step is detailed with visual screenshots from the software for both control and transduced cells.

Step 1: Input Image

- Example images of control and αSyn fibril-transduced cells are loaded into the software.

Step 2: Find Nuclei/Channel 445/45 (Method M)

- The script identifies the nuclei in both control and transduced cells. Nuclei detection is visualized and highlighted in both sets of cells.

Step 3: Find Spots/Channel BP525/50 (Method C)

- In this step, the script detects CFP/YFP inclusions (spots) in the cells, differentiating between control and transduced conditions.

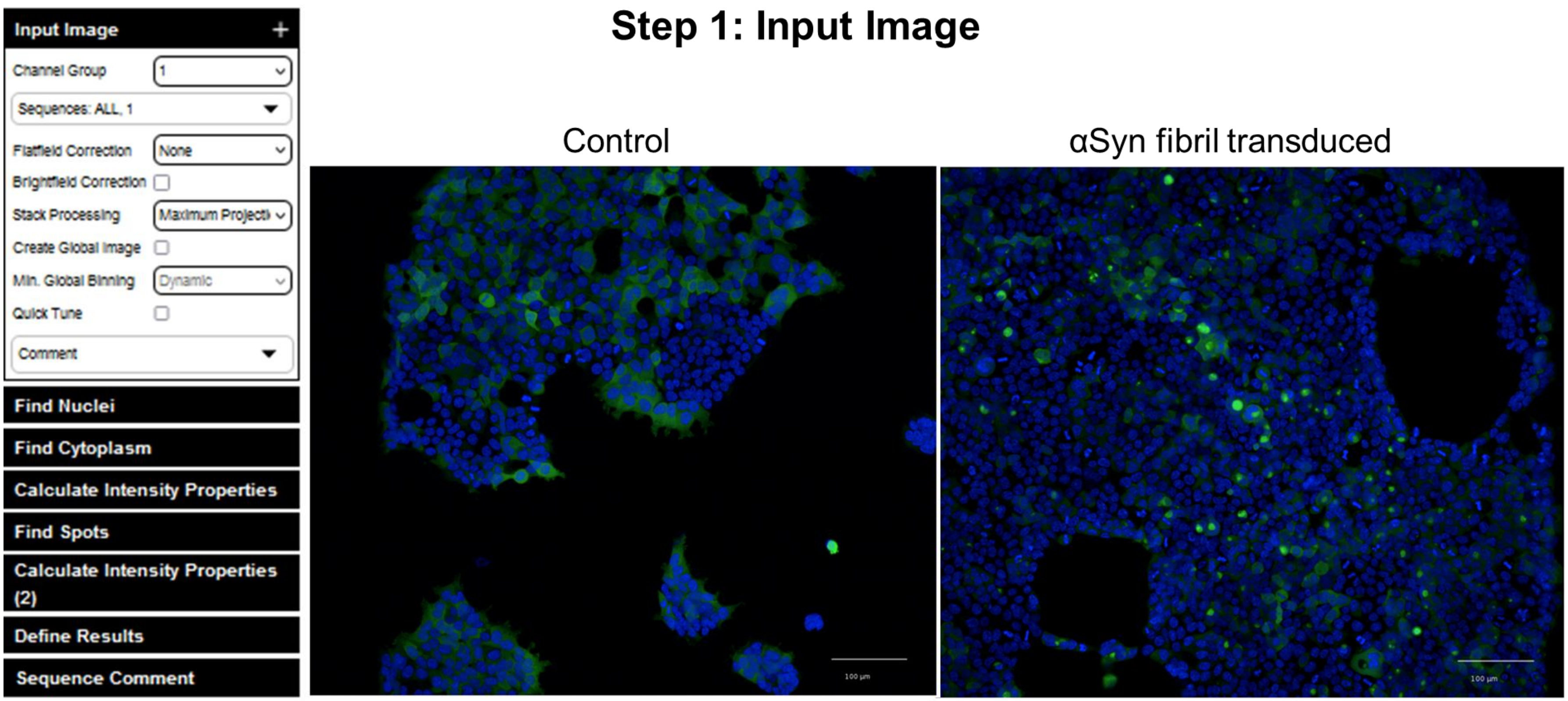
Step 1: Input Image
Input Image
Channel Group
1
Sequences: ALL, 1
Flatfield Correction
None
Brightfield Correction
Stack Processing
Maximum Projecti
Create Global Image
Min. Global Binning
Dynamic
Quick Tune
Comment
Find Nuclei
Find Cytoplasm
Calculate Intensity Properties
Find Spots
Calculate Intensity Properties (2)
Define Results
Sequence Comment
Control
αSyn fibril transduced
100 µm
100 µm

Step 2: Find Nuclei/Channel 445/45 (Method M)

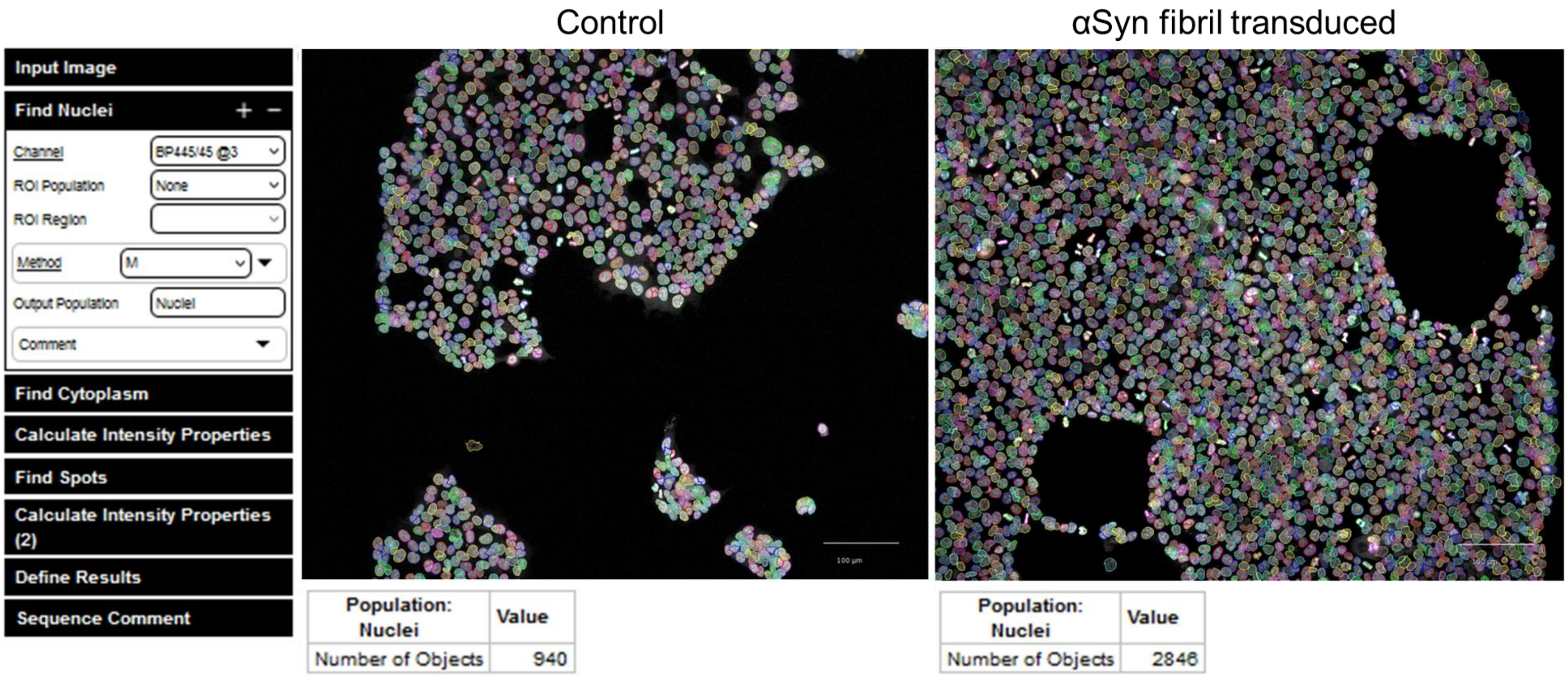

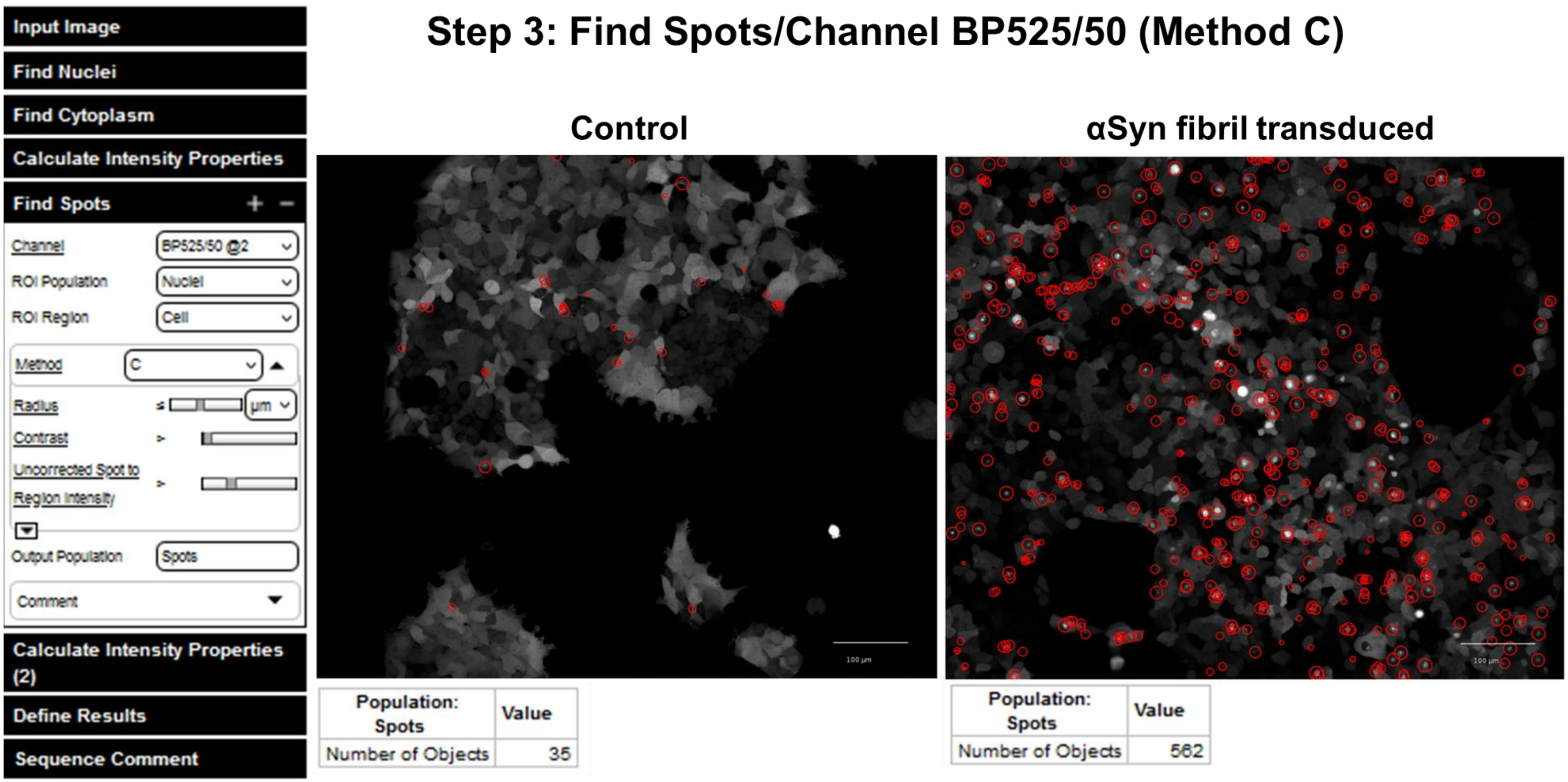
Input Image
Find Nuclei
Find Cytoplasm
Calculate Intensity Properties
Find Spots
Channel
BP525/50 @2
ROI Population
Nuclei
ROI Region
Cell
Method
C
Radius
µm
Contrast
Uncorrected Spot to Region Intensity
Output Population
Spots
Comment
Calculate Intensity Properties (2)
Define Results
Sequence Comment
Step 3: Find Spots/Channel BP525/50 (Method C)
Control
αSyn fibril transduced
Population: Spots
Value
Number of Objects
35
Population: Spots
Value
Number of Objects
562
